# Connexin 30 Deficiency Attenuates Chronic but Not Acute Phases of Experimental Autoimmune Encephalomyelitis Through Induction of Neuroprotective Microglia

**DOI:** 10.3389/fimmu.2018.02588

**Published:** 2018-11-07

**Authors:** Mei Fang, Ryo Yamasaki, Guangrui Li, Katsuhisa Masaki, Hiroo Yamaguchi, Atsushi Fujita, Noriko Isobe, Jun-ichi Kira

**Affiliations:** ^1^Department of Neurology, Neurological Institute, Graduate School of Medical Sciences, Kyushu University, Fukuoka, Japan; ^2^Department of Neurological Therapeutics, Neurological Institute, Graduate School of Medical Sciences, Kyushu University, Fukuoka, Japan

**Keywords:** astrocyte, chronic neuroinflammation, connexin, experimental autoimmune encephalomyelitis, microglia, multiple sclerosis

## Abstract

Glial connexins (Cxs) form gap junction channels through which a pan-glial network plays key roles in maintaining homeostasis of the central nervous system (CNS). In multiple sclerosis (MS) and its animal model, experimental autoimmune encephalomyelitis (EAE), expression of astrocytic Cx43 is lost in acute lesions but upregulated in chronic plaques, while astrocytic Cx30 is very low in normal white matter and changes in its expression have not been convincingly shown. In Cx30 or Cx43 single knockout (KO) mice and even in Cx30/Cx43 double KO mice, acute EAE is unaltered. However, the effects of Cx30/Cx43 deficiency on chronic EAE remains to be elucidated. We aimed to clarify the roles of Cx30 in chronic neuroinflammation by studying EAE induced by myelin oligodendrocyte glycoprotein peptide 35–55 in Cx30 KO mice. We found that Cx30 deficiency improved the clinical symptoms and demyelination of chronic but not acute EAE without influencing CD3^+^ T cell infiltration. Furthermore, increased ramified microglia in the naïve state and induced earlier and stronger microglial activation in the acute and chronic phases of EAE was observed. These activated microglia had an anti-inflammatory phenotype, as shown by the upregulation of arginase-1 and brain-derived neurotrophic factor and the downregulation of nitric oxide synthase 2. In the naïve state, Cx30 deficiency induced modest enlargement of astrocytic processes in the spinal cord gray matter and a partial reduction of Cx43 expression in the spinal cord white matter. These astrocytes in Cx30 KO mice showed earlier and stronger activation during the acute phase of EAE, with upregulated A2 astrocyte markers and a significant decrease in Cx43 in the chronic phases. Spinal cord neurons and axons were more preserved in Cx30 KO mice than in littermates in the chronic phase of EAE. These findings suggest that Cx30 deficiency increased ramified microglia in the CNS in the naïve state and improved chronic EAE through redirecting microglia toward an anti-inflammatory phenotype, suggesting a hitherto unknown critical role of astrocytic Cx30 in regulating microglial number and functional state.

## Introduction

Multiple sclerosis (MS) is an inflammatory demyelinating disease of the central nervous system (CNS) (1). It initially presents as relapsing remitting MS (RRMS) but later evolves into secondary progressive MS (SPMS) in ~20% of patients, even after disease-modifying therapies (DMTs) are introduced (2, 3). Most DMTs, mainly targeting the peripheral immune system, can effectively reduce relapses in RRMS; however, they are of little benefit for chronic progression in SPMS (4–6). Thus, chronic progression in MS is currently a matter of concern for research and drug development. Recently, siponimod (7) and ozanimod (8), new functional antagonists of sphingosine-1-phosphate receptor 1 (S1P1), were reported to be effective for preventing disability progression in SPMS. These drugs may directly act on glial cells harboring S1P1 such as microglia and astroglia, in addition to their inhibitory effects on lymphocyte egress from the secondary lymphoid organs (9–11). In chronic MS lesions, persistent demyelination with varying degrees of remyelination and neuroaxonal degeneration are accompanied by the presence of activated microglia but few T cells (12, 13), suggesting a key role of microglia, which are not targeted by the peripherally acting DMTs, in chronic inflammation in SPMS.

Connexins (Cxs) form gap junction (GJ) channels, which allow the intercellular exchange of ions and secondary messengers (14). In the CNS, astrocytes express Cx43, Cx30, and Cx26, while oligodendrocytes express Cx47, Cx32, and Cx29 (15–17). These Cxs constitute a pan-glial network through GJ channels and play key roles in maintaining CNS homeostasis (18–20). We and others have reported dynamic changes of glial Cxs in MS and Baló's concentric sclerosis lesions (21–23). Oligodendrocytic Cx47 and Cx32 are persistently lost in acute and chronic MS plaques, while astrocytic Cx43 is lost in acute lesions and then upregulated in chronic astrogliotic plaques (22–24). Similar changes in Cx47, Cx32, and Cx43 were also observed in acute and chronic experimental autoimmune encephalomyelitis (EAE), an animal model of MS (25–28). These findings suggest the involvement of glial Cxs in inflammatory demyelination.

Consistent with this notion, oligodendrocytic Cx32 knockout (KO) mice developed aggravated acute and chronic EAE, with increased demyelination despite a similar degree of inflammation upon immunization with myelin oligodendrocyte glycoprotein (MOG), compared with wild type (WT) mice (25). By contrast, in astrocytic Cx30 or Cx43 single KO mice and even in Cx30/Cx43 double KO mice, acute EAE was unaltered (29). However, it remains to be elucidated whether a deficiency in Cx30 or Cx43 influences chronic EAE.

Because the expression level of astrocytic Cx30 is very low in normal white matter, changes of Cx30 in MS or EAE lesions have not been well demonstrated (22, 23, 30). Cx43 exists in both mature and immature astrocytes, while Cx30 is expressed only in mature astrocytes (31–33), thus gliotic scar astrocytes show an upregulation of Cx43 but no detectable changes of Cx30 (23). Similarly, cultured astrocytes express detectable levels of Cx43 but not Cx30, although they can express Cx30 after very long term culture (33, 34). These features of Cx30 make it difficult to study its dynamics and roles in inflammatory demyelination, and therefore there have been few studies of Cx30 in EAE. However, the non-channel functions of Cxs have recently gained increasing attention: Cx30 can change astrocyte morphology, thereby modulating astrocyte functions such as synaptic transmission (35). Cxs also inhibits DNA synthesis, which affects the gene expression network (36, 37).

In the present study, we aimed to clarify the roles of Cx30 in chronic neuroinflammation by studying chronic EAE in Cx30 KO mice. Here, we report Cx30 deficiency induces anti-inflammatory microglia and improves clinical symptoms and demyelination of chronic but not acute EAE.

## Materials and methods

### Ethics statement

The experimental procedures were designed to minimize the number of animals used as well as animal suffering. All animal experiments were carried out according to the guidelines for proper conduct of animal experiments published by the Science Council of Japan and the ARRIVE (Animal Research: Reporting of *in vivo* Experiments) guidelines for animal research. Ethical approval for the study was granted by the Animal Care and Use Committee of Kyushu University (#A29-146-3).

### Animals and genotyping

Twelve-to-sixteen-week-old female Cx30 KO mice were used in this study. Cx30 KO mice (38) that had been backcrossed to C57BL/6J at the archiving center were purchased from the European Mouse Mutant Archive. C57BL/6 mice were purchased from KBT Oriental (Tosu, Japan). All mice were bred and maintained under specific pathogen free conditions in the Center of Biological Research, Graduate School of Medical Sciences, Kyushu University. The Cx30 KO mice were genotyped by PCR of DNA obtained from tail biopsies. Primer pairs for detecting Cx30 KO were Cx30 KO-1 (LACZ e Neo): 5′-GGT ACC TTC TAC TAA TTA GCTTGG-3′; Cx30 KO2 (LACZ e Neo): 5′-AGG TGG TAC CCA TTG TAG AGG AAG-3′; and Cx30 KO-3 (LACZ e Neo): 5′-AGC GAG TAA CAA CCC GTC GGA TTC-3′. The Cx30 KO and WT littermate DNA products were 460 and 544 bps in size, respectively. Cx30 KO mice and their littermates were principally used for the animal experiments, unless otherwise specified.

### Induction and clinical evaluation of EAE

EAE was induced by immunization of mice with 200 μg of MOG_35−55_ peptide (TS-M704-P; MBL, Nagoya, Japan) in 50 μl phosphate buffered saline (PBS) emulsified in an equal volume of complete Freund's adjuvant (CFA) containing 1 mg/ml *Mycobacterium tuberculosis* H37RA (#231131; BD Difco, Lawrence, KS, USA), followed by intraperitoneal injections of 500 ng pertussis toxin (# 180-A1; List Biological Laboratories Inc., Campbell, CA, USA) on days 0 and 2. Mice were examined daily for signs of EAE and scored as follows: 0, no disease; 1, limp tail; 2, abnormal gait and hind limb weakness (shaking); 2.5, paralysis of one hind limb; 3, paralysis of two hind limbs; 3.5, ascending paralysis (able to move around); 4, tetraplegia; 5, moribund.

### Tissue preparation

Animals were deeply anesthetized by isoflurane (Pfizer Japan Inc., Tokyo, Japan), and perfused transcardially with PBS and then with 4% paraformaldehyde (PFA) in 0.1 M PBS. Spinal cords, brains and optic nerves were carefully dissected. The tissues were fixed overnight in cold 4% PFA at 4°C, then processed into paraffin sections (5 μm). For frozen sections (20 μm), spinal cords were harvested and fixed overnight in 4% PFA using the same protocol as above and sequentially displaced with 15 and 30% sucrose in PBS for 24 h each at 4°C. The resulting tissues were embedded in Tissue-Tek O.C.T. Compound (4583, Sakura Finetek, Torrance, CA, USA) and stored at −80°C.

### Histopathological and immunohistochemical analyses

Paraffin-embedded sections of spinal cord were stained with hematoxylin and eosin (HE). Paraffin-embedded sections of optic nerves were subjected to immunohistochemistry using an indirect immunoperoxidase method. After deparaffinization, endogenous peroxidase was quenched with 0.3% hydrogen peroxide in absolute methanol for 30 min. The sections were permeabilized with 0.1% Triton in PBS (PBS-T) for 10 min, washed using Tris-HCl for 5 min, dipped in 10 mM citrate buffer, and then autoclaved (120°C, 10 min). All sections were cooled to room temperature and incubated with anti-brain-derived neurotrophic factor (BDNF) antibodies overnight at 4°C (Supplementary Table 1). The next day, after rinsing, sections were labeled with either a streptavidin-biotin complex or an enhanced indirect immunoperoxidase method using Envision (K4003, Dako, Glostrup, Denmark); 3,3-diaminobenzidine tetrahydrochloride (DAB; D5637, Sigma-Aldrich, Tokyo, Japan) was used for the DAB color reaction. Finally, sections were counterstained with hematoxylin.

### Confocal microscope immunofluorescence analysis

Paraffin sections of brain and optic nerves were deparaffinized in xylene and rehydrated through ethanol. After washing and autoclaving, sections were incubated with anti-arginase1, anti-nitric oxide synthase 2 (NOS2), anti-Iba-1, anti-glial fibrillary acidic protein (GFAP), anti-Cx43, anti-Cx30, anti-myelin basic protein (MBP), anti-NeuN, purified anti-neurofilament H (NF-H) (SMI-31), and anti-IL-34 antibodies (Supplementary Table 1) overnight at 4°C. The following day, the sections were washed, incubated with Alexa Fluor 488- or 546-conjugated secondary antibodies (1:1,000; Thermo Fisher, Rockford, IL, USA) and 4′,6-diamidino-2-phenylindole (DAPI; Sigma-Aldrich, Tokyo, Japan) overnight at 4°C, then dehydrated and sealed with Permafluor (#TA-030-FM; Thermo Scientific, Fremont, CA, USA). The frozen sections of spinal cords were cut at 20 μm with a cryostat microtome (Leica CM 1850, Leica Microsystems GmbH, Wetzlar, Germany) and floated in PBS-T. The sections were washed 3 times in PBS-T, blocked with 10% normal goat serum in PBS for 2 h, then incubated overnight at 4°C with anti-arginase1, anti-NOS2, anti-Cx30, anti-Cx43, anti-Iba-1, anti-GFAP, anti-CD45, anti-CD3, anti-C3, and anti-CD169 antibodies (Supplementary Table 1). The sections were also treated with anti-S100a10 antibody in the same way but without blocking by normal goat serum (Supplementary Table 1). After rinsing the next day, the sections were incubated with Alexa Fluor 488- or 546-conjugated secondary antibodies (1:1,000; Thermo Fisher) or FluoroMyelin Red Fluorescent Myelin Stain (1:1,000; #F34652; Thermo Fisher) and DAPI overnight at 4°C, then washed in PBS-T and sealed with Permafluor. Immunofluorescence was captured by a confocal laser microscope (Nikon A1; Nikon, Tokyo, Japan), equipped with 405, 488, and 561 nm laser lines, at the same magnification, laser intensity, gain, offset values, and pinhole settings. Quantification of immunofluorescence was performed using ImageJ version 1.6.0_24 (Windows version of NIH Image; downloaded from https://imagej.nih.gov/ij/download.html) on three-to-five lumbar spinal cord sections for each animal in each group.

### Quantification of myelin density and cell infiltration in the spinal cord, brain, and optic nerve

For the quantification of GFAP, Iba-I, Cx30, Cx43, CD3, CD169, CD45, S100A10, C3, BDNF, NOS2, and IL-34, fluorescent images from the anterior part of the lumbar spinal cord, cerebellum, cerebrum, and optic nerve were analyzed (ImageJ version 1.6.0_24) using the area fraction technique as previously described (39, 40). Briefly, identical microscope settings were applied to all photographs from each experiment and images from the same areas were acquired. Images were de-noised and set to the same threshold baseline across experimental groups for each antibody to measure the area of cellular staining, instead of cell density measurement or cell number counting, because most infiltrating cells were focally clustered. For the quantification of myelin and MBP immunostaining results, whole spinal cord images were captured under the microscope and separated into anterior or posterior parts for analysis. SMI-31 immunostaining images were captured under the microscope and spinal cord anterior white matter areas were used for analysis. Image analysis was performed using ImageJ software. Mean pixel intensity values were compared between genotypes (41). For the quantitative analyses of NeuN-positive cells, transverse sections of the spinal cord were divided into the left and right regions by a vertical line through the central canal. The size of each microscopic field was 1.6384 mm^2^. The left or right positive cell region areas (0.33–0.44 mm^2^) were calculated automatically by ImageJ. NeuN-positive cells were counted manually and used to calculate the cell density (neurons/mm^2^) (42). The investigator performing the analysis was blinded to the genotypes. All assessments were made from three-to-five sections per mouse (*n* = 3 to 8 mice in each group). In the quantification graph, the mean value of three-to-five sections was used as scatter dots to represent each mouse.

### Immunocolocalization analysis

We delineated the same areas of focus in the spinal cord white matter, optic nerve, and arbor vitae of the cerebellum in all samples to be analyzed. Colocalization of arginase-1 and Iba-1 was expressed as a Pearson's correlation coefficient and the intensity correlation analysis plugin of ImageJ was used (43). Pearson's correlation values range from 1 to −1, with 1 representing complete positive correlation and −1 a negative correlation, with zero representing no correlation. All quantifications were obtained from a minimum of three sections from the spinal cord, optic nerve, cerebellum, and cerebrum per mouse.

### Microglial circularity analysis

ImageJ was used to automatically calculate the circularity of microglial cells (circularity = 4πS/L2). Cells with circularity close to 1 were regarded as having a morphology close to round, indicating an activated state (44, 45).

### Microglial cell isolation and flow cytometry

Brains and spinal cords were harvested and homogenized. Mononuclear cells were separated with a 30 and 70% Percoll (GE Healthcare, Tokyo, Japan) gradient as previously described (46). Cells were stained with anti-CD45-PerCP and anti-CD11b-PE/Cy7 antibodies, sorted and analyzed on a SH800 Cell Sorter (Sony Corporation, Tokyo, Japan).

### Gene expression microarray

Total RNA was isolated from cells using an RNeasy Mini Kit (Qiagen) according to the manufacturer's instructions. RNA samples were quantified by an ND-1000 spectrophotometer (NanoDrop Technologies, Wilmington, DE, USA) and the quality was confirmed with a 2200 TapeStation (Agilent technologies, Santa Clara, CA, USA). Total RNA (2 ng) was amplified, labeled using a GeneChip® WT Pico Kit, and hybridized to an Affymetrix GeneChip® Mouse Transcriptome Array 1.0 according to the manufacturer's instructions (Affymetrix, Santa Clara, CA, USA). All hybridized microarrays were scanned by an Affymetrix scanner. Relative hybridization intensities and background hybridization values were calculated using the Affymetrix Expression Console®. These gene array assay results were uploaded to the gene expression omnibus repository (accession number is GSE68202) in the National Center for Biotechnology Information homepage (https://www.ncbi.nlm.nih.gov/geo/query/acc.cgi?acc=GSE112621).

### Data analysis and filter criteria

The raw signal intensities of all samples were normalized by a quantile algorithm with Affymetrix® Power Tool version 1.15.0 software. To identify upregulated or downregulated genes, we calculated Z-scores [Z] and ratios (non-log scaled fold-change) from the normalized signal intensities of each probe for comparison between control and experiment samples. Then, we established criteria for regulated genes: upregulated genes had a Z-score ≥2.0 and ratio ≥1.5-fold, and downregulated genes had a Z-score ≤ −2.0 and ratio ≤ 0.66. Gene set enrichment analysis (47, 48) (GSEA; www.broadinstitute.org/gsea) was performed to investigate deviations of particular gene sets (e.g., Anti-inflammatory set, Pro-inflammatory set; set S) according to a previous report (48, 49). Briefly, after we obtained expression data sets for each study group, we calculated an enrichment score (ES) that reflected the degree to which a set “S” was over-represented at the extremes (top or bottom) of the entire ranked list “L.” The score was calculated by walking down the list L and increasing a running-sum statistic when we encountered a gene in S. The ES is the maximum deviation from zero encountered in the random walk. After the estimation of statistical significance of ES, we controlled the proportion of false positives by calculating the false discovery rate (FDR). When the normalized *p*-value was < 0.05 and the FDR was < 0.25, the ES was considered significant.

### Statistical analysis

Data are expressed as the mean ± standard error of mean (S.E.M.). The area under curve (AUC) of the overall disease severity was calculated for each mouse to compare the disease course of WT and KO mice using the non-parametric Mann-Whitney *U*-test (50) Here, acute (onset to day 24) and chronic phases (day 25 and thereafter) were separately analyzed. The postimmunization date when WT groups reached a peak score of 2 or higher was identified as the “peak” (51). The incidence, day of onset, and peak clinical score of EAE were compared using the unpaired *t-*test with Welch's correction. In EAE experiments, mice that died before the intended day of sacrifice were excluded from statistical analyses. Cell percentages and histological data were assessed by the unpaired *t*-test with Welch's correction, two-way ANOVA, or one-way ANOVA. A *p*-value < 0.05 was considered statistically significant. Analyses were performed using Graph Pad Prism 7.0 software (Graph Pad, La Jolla, CA, USA).

## Results

### Cx30 deficiency induces modest morphological changes and Cx43 reduction in spinal cord astrocytes but no changes in myelin density

**Figure 1:**
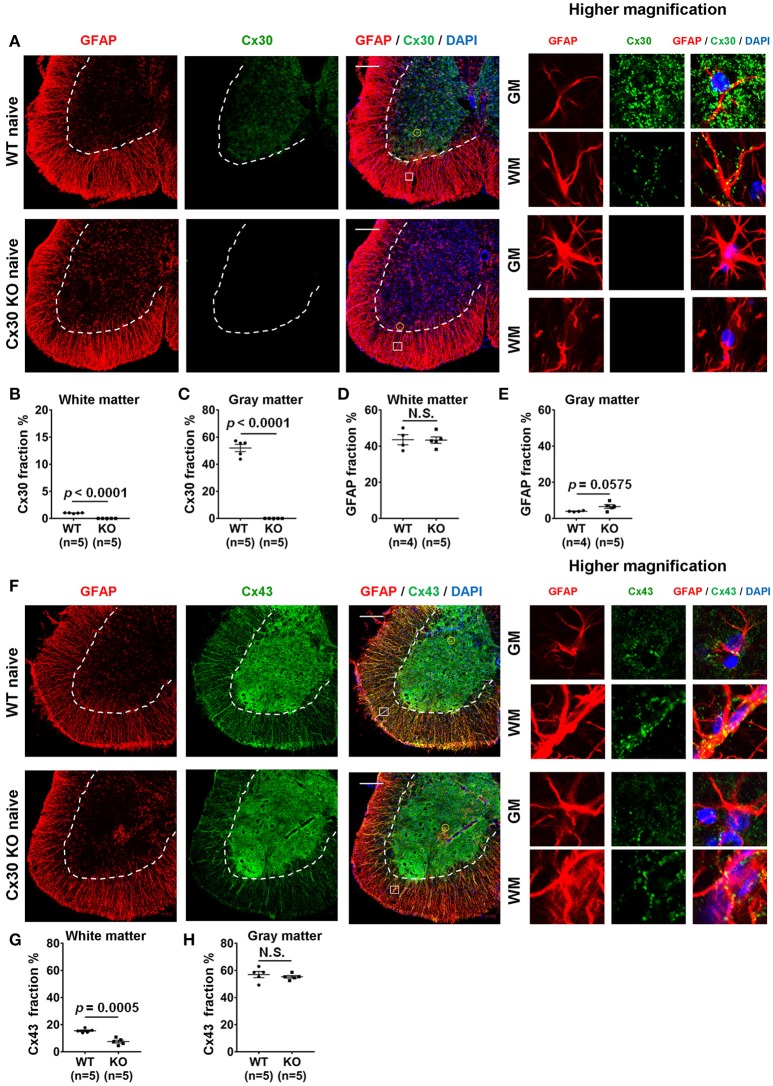
Morphology and number of astrocytes, and expressions of Cx30 and Cx43 in WT and Cx30 KO mice. **(A)** Confocal images showing immunostaining for Cx30 and GFAP in the anterior part of spinal cord sections from naïve WT (littermate) and Cx30 KO mice. Higher magnification images show co-labeling of GFAP and Cx30 in single astrocytes, which are highlighted by a yellow circle in the gray matter or a white square in the white matter of each figure. Scale bars, 200 μm. **(B,C)** Quantification of Cx30^+^ area percentages in the white **(B)** and gray **(C)** matter. **(D,E)** Quantification of GFAP^+^ area percentages in the white **(D)** and gray **(E)** matter. **(F)** Confocal images showing immunostaining for GFAP and Cx43 in spinal cords from naïve WT (littermate) and Cx30 KO mice. Higher magnification images show co-labeling of GFAP and Cx43 in single astrocytes, which are highlighted by a yellow circle in the gray matter or a white square in the white matter of the figure. Scale bars, 200 μm. **(G,H)** Quantification of Cx43^+^ area percentages in the white matter **(G)** and gray matter **(H)**. Means ± S.E.M. are shown. Statistical differences were determined by unpaired *t-*test with Welch's correction. N.S. = not significant, *n* indicates number of mice and each scatter dot represents individual mice in each group.

In WT littermate mice in the naïve state, Cx30 was expressed predominantly on astrocytes in the gray matter of the spinal cord, cerebellum and cerebrum, while Cx30 expression was very low in white matter astrocytes, including the optic nerve (Figure 1A; Supplementary Figure 1), which is consistent with our previous study in humans (22). By contrast, Cx30 was completely absent in Cx30 KO mice (Figures 1A–C; Supplementary Figure 1). GFAP immunostaining revealed neither morphological nor quantitative changes in GFAP^+^ astrocytes in the white matter between WT and Cx30 KO mice, whereas GFAP^+^ astrocytes in the gray matter had thicker processes and showed a tendency to be increased in Cx30 KO mice than in WT mice (*p* = 0.0575, Figures 1A,D,E).

Because astrocytic Cx30 and Cx43 have similar functions and partly overlapping permeation profiles (15), we examined whether Cx43 was upregulated to compensate for the lack of Cx30 in Cx30 KO mice. GFAP and Cx43 double immunostaining revealed that Cx43 was more abundant in the gray matter than in the white matter of the spinal cord in both WT and Cx30 KO mice (Figures 1F–H). There was no significant difference in Cx43 immunoreactivity in the spinal cord gray matter between WT and Cx30 KO mice, but Cx43 levels were significantly reduced in the spinal cord white matter of Cx30 KO mice compared with WT mice (*p* = 0.0005; Figures 1F–H). These findings are consistent with the observation that Cx30 but not Cx43 can compensate for other Cxs in CNS tissues (52). This suggests that Cx30 deficiency causes modest enlargement of astrocytic processes in the spinal gray matter and a partial reduction of Cx43 expression in spinal white matter astrocytes. However, myelin density did not differ significantly between Cx30 KO mice and WT littermates in the naive state by Fluoromyelin staining or MBP immunostaining (Supplementary Figures 2A–D).

### Cx30 deficiency increases the numbers of ramified microglia

**Figure 2:**
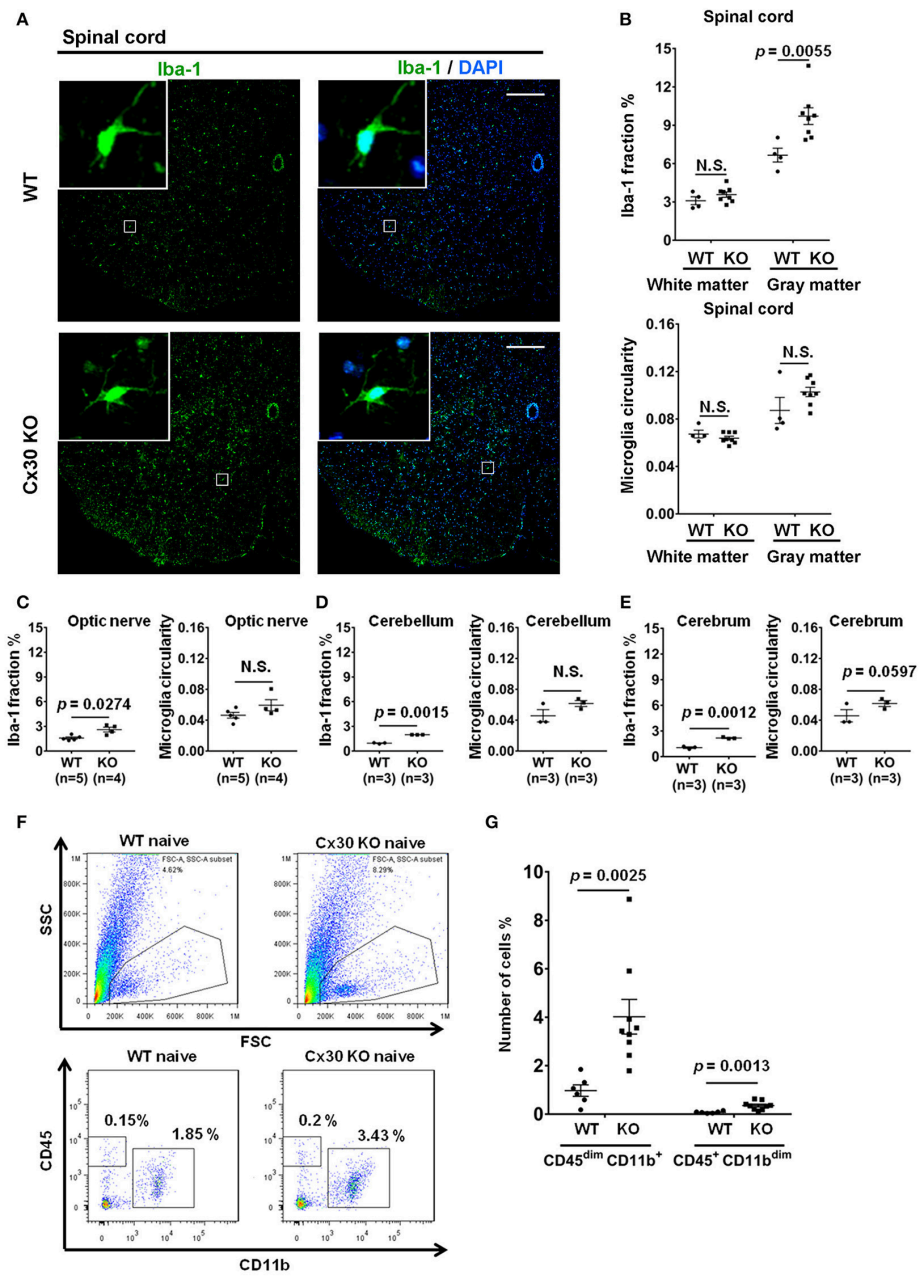
Microglial morphology and numbers in CNS tissues of WT and Cx30 KO mice. **(A)** Representative images of Iba-1 immunostaining of naïve WT (littermate) and Cx30 KO mouse spinal cord. Scale bars, 200 μm. **(B–E)** Quantification of Iba-1^+^ cell area fractions and microglia circularity in the white and gray matter of the anterior spinal cord **(B)**, optic nerve **(C)**, cerebellum **(D)**, and cerebrum **(E)**. **(F)** Gating strategy used to determine viable mononuclear cells for further analysis. In the whole brain suspension, a gate was created on the non-debris population. Inside this population, the microglial cells were gated based on CD45/CD11b intensity. SSC, Side scatter; FSC, Forward scatter. Representative flow cytometric analysis of microglia (CD45^dim^ CD11b^+^ cells) isolated from WT (littermate) and Cx30 KO mouse brains. Numbers on plots are percentages of double-positive cells among the gated viable cells. **(G)** Percentages of CD45^dim^ CD11b^+^ microglia and CD45^+^ CD11b^dim^ cells in the total cell population (3 × 10^4^) isolated from naïve WT (littermate) and Cx30 KO mouse brains. Data are from 6 WT mice and 9 Cx30 KO mice. Means ± S.E.M. are shown. Statistical differences were determined using the unpaired *t-*test with Welch's correction. N.S. = not significant. *n* indicates the number of mice and each scatter dot represents individual mice in each group.

Unexpectedly, Cx30 KO mice showed an apparent increase in the numbers of Iba-1^+^ microglia in the spinal cord gray matter, optic nerve, cerebellum, and cerebrum compared with WT littermate mice (*p* = 0.0055, *p* = 0.0274, *p* = 0.0015, and *p* = 0.0012, respectively), but not in the spinal cord white matter (Figures 2A–E). Morphologically, microglia in Cx30 KO mice had thin soma and delicate radially-projecting processes (Figure 2A inset), indicating that these microglia were in a resting state (ramified phenotype). There were no significant differences in the microglia circularity index between Cx30 KO and WT mice in the spinal cord gray and white matter, optic nerve, cerebrum, and cerebellum (Figures 2B–E). Flow cytometric analyses demonstrated that numbers of CD45^dim^CD11b^+^ microglia in isolated viable cells from the brain were significantly increased in Cx30 KO mice compared with WT littermate mice (*p* = 0.0025; Figures 2F,G).

**Figure 3:**
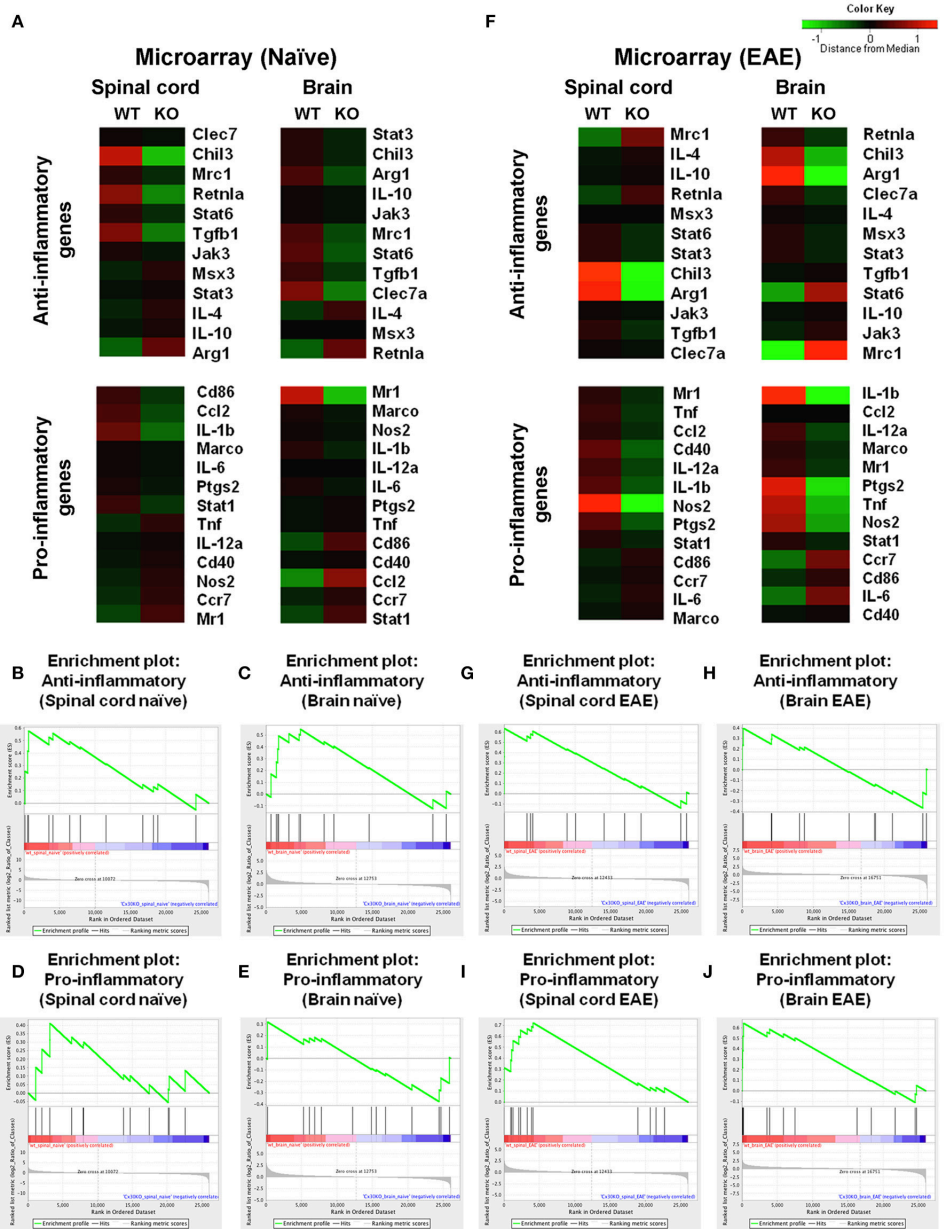
Microarray analysis of microglia isolated from spinal cords and brains of WT (C57BL/6) and Cx30 KO mice. **(A)** Cluster analysis of gene expression arrays according to anti-inflammatory and pro-inflammatory genes of naïve brain and spinal cord from WT (C57BL/6) and Cx30 KO mice. Color keys on each column represent *Z* scores for each gene. **(B–E)** Enrichment plots for the anti-inflammatory **(B,C)** and pro-inflammatory **(D,E)** genes of naive spinal cords **(B,D)** and brains **(C,E)** from WT (C57BL/6) and Cx30 KO mice. **(F)** Cluster analysis of gene expression arrays according to anti-inflammatory and pro-inflammatory genes of chronic EAE brains and spinal cords from WT (C57BL/6) and Cx30 KO mice. Color keys on each column represent *Z* scores for each gene. **(G–J)** Enrichment plots for the anti-inflammatory **(G,H)** and pro-inflammatory **(I,J)** genes of chronic EAE spinal cords **(G,I)** and brains **(H,J)** from WT (C57BL/6) and Cx30 KO mice. The relative gene positions are indicated by the straight lines (line plot) under each graph. Lines clustered to the left represent higher ranked genes in the ranked list.

To further characterize the microglial phenotype in Cx30 KO mice, gene expression profiles were analyzed by RNA microarray using microglia isolated from the spinal cords and brains of naïve WT and Cx30 KO mice. Microglia from Cx30 KO mice showed similar expression levels of anti-inflammatory and pro-inflammatory genes to WT microglia in both the spinal cord and brain (Table 1 and Figure 3A). GSEA analysis revealed similar gene enrichments in the spinal cord and brain between naive WT and KO mice (Table 1 and Figures 3B–E). We also performed GSEA analysis to characterize the expression profiles of cytokines/chemokines, complement, alarmin, reactive oxygen species (ROS), MHC, and tumor genes. Among them, Cx30 KO microglia from the naïve spinal cord but not brain demonstrated significantly lower expression levels of cytokines/chemokines, alarmin, MHC, and tumor genes, indicating a less reactive state to inflammatory insults (Table 1 and Supplementary Figures 3A,C). These findings indicate that the increase in microglia was widespread in the CNS of naïve Cx30 KO mice compared with WT mice; these microglial were not activated but rather in a resting state, with a ramified morphology and low cytokine/chemokine, alarmin, MHC, and tumor gene production.

**Table 1:** Summary of GSEA results.

**Gene category**	**Naive spinal cord**	**EAE spinal cord**	**Naive brain**	**EAE brain**
	**WT vs. Cx30 KO**	**WT vs. Cx30 KO**	**WT vs. Cx30 KO**	**WT vs. Cx30 KO**
Pro-inflammatory	0.392 (WT)	0.007 (WT)	0.571 (Cx30 KO)	0.016 (WT)
Anti-inflammatory	0.083 (WT)	0.092 (WT)	0.234 (WT)	0.450 (WT)
Cytokines/Chemokines	<0.001 (WT)	<0.001 (WT)	0.773 (WT)	0.252 (Cx30 KO)
Complement	0.997 (Cx30 KO)	0.001 (WT)	0.576 (WT)	0.348 (Cx30 KO)
Alarmin	<0.001 (WT)	0.005 (WT)	0.898 (Cx30 KO)	0.061 (WT)
ROS	0.524 (WT)	0.005 (WT)	0.093 (Cx30 KO)	0.363 (Cx30 KO)
MHC	<0.001 (WT)	0.008 (WT)	0.173 (WT)	<0.001 (Cx30 KO)
Tumor	<0.001 (WT)	<0.001 (WT)	0.967 (Cx30 KO)	0.001 (Cx30 KO)

*Normalized p-values by gene set enrichment analysis between WT (C57BL/6) and Cx30 KO mice are shown. Upregulated groups are indicated in parenthesis for each category*.

### Cx30 deficiency attenuates the clinical severity and demyelination of chronic but not acute EAE without influencing T cell infiltration

**Figure 4:**
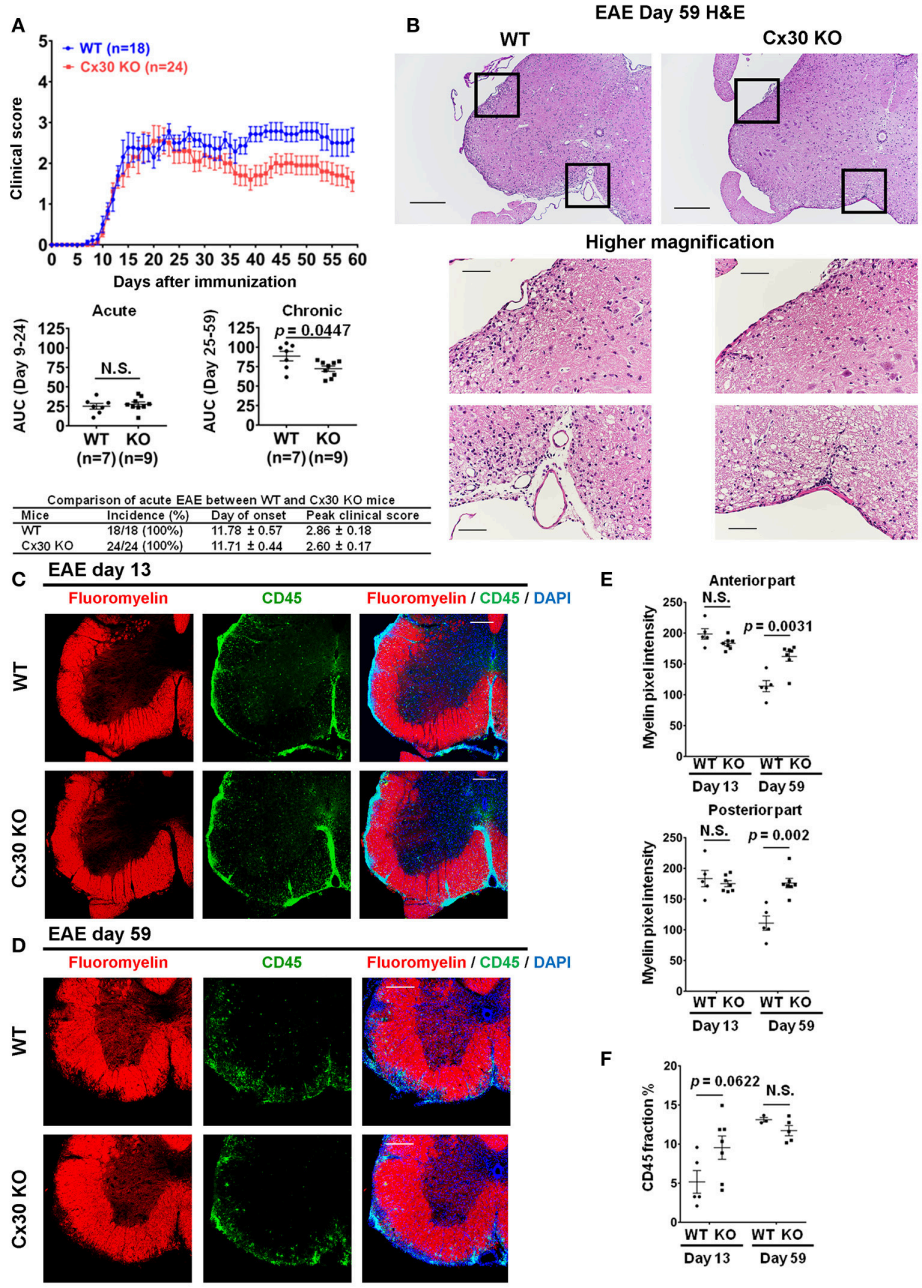
Improvement of clinical severity and demyelination in the chronic but not acute phase of EAE in Cx30 KO mice. **(A)** EAE clinical score changes in WT (littermate) and Cx30 KO mice. The severity of disease was separately analyzed according to acute (Days 9–24) and chronic (Days 25–59) phases by evaluating the area under the curve (AUC). Data shown are from a single experiment representative of four independent experiments including a total of 42 mice; *p*-values of the AUC were determined by the Mann-Whitney *U*-test. There was no significant difference in incidence, day of onset, or peak clinical score between WT (littermate) and Cx30 KO mice in the acute EAE phase. Data for the following parameters are shown as the mean ± S.E.M.: day of EAE onset, and peak clinical score of mice that developed EAE. Statistical differences were determined using the unpaired *t*-test with Welch's correction. **(B)** HE staining of spinal cords in the chronic EAE phase (Day 59). Scale bars, 200 μm in the upper panels and 100 μm in the lower panels. **(C,D)** Confocal images showing immunostaining for Fluoromyelin and CD45 in spinal cord sections from WT (littermate) and Cx30 KO mice in the acute (Day 13) and chronic (Day 59) EAE phases. Scale bars, 200 μm. **(E)** Quantification of myelin density in the anterior and posterior parts of spinal cords from WT (littermate) and Cx30 KO mice in the acute (Day 13) and chronic (Day 59) EAE phases. **(F)** Quantification of the CD45^+^ cell area fraction in the anterior spinal cords from WT (littermate) and Cx30 KO EAE mice in the acute (Day 13) and chronic EAE phases (Day 59). Statistical differences were determined using the unpaired *t-*test with Welch's correction. N.S. = not significant. *n* indicates the number of mice and each scatter dot represents individual mice in each group.

**Figure 5:**
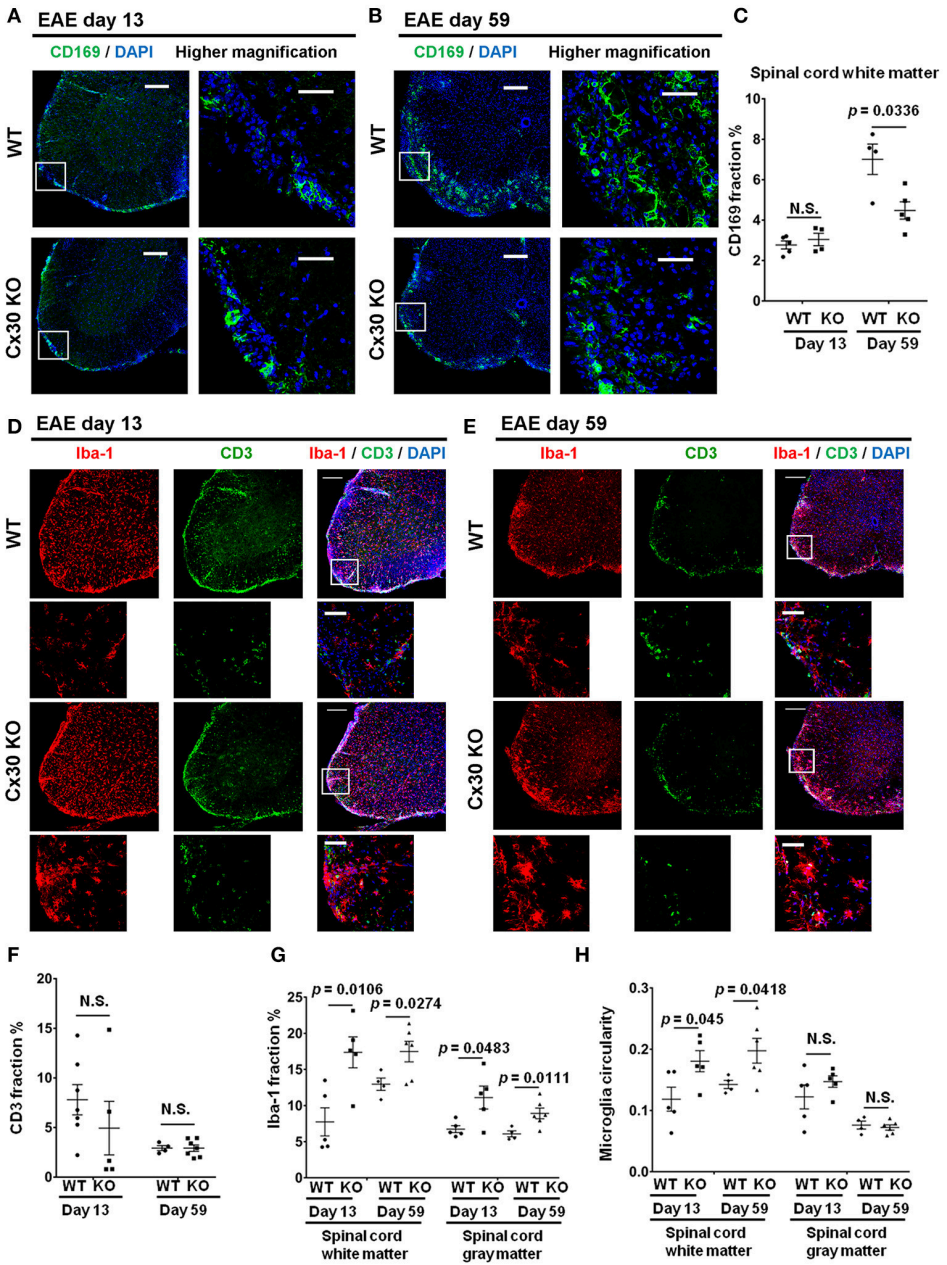
Altered immune cell responses in Cx30 KO mice in the chronic EAE phase compared with WT mice. **(A,B)** Confocal images showing immunostaining of CD169 in spinal cord sections from WT (littermate) and Cx30 KO EAE mice in the acute (Day 13) and chronic (Day 59) EAE phase. Scale bars, 200 μm. Higher magnification images are CD169 in the spinal cord, which are highlighted by a white rectangular frame in the left panel of lower magnification images. **(C)** Quantification of the CD169^+^ cell area fraction in the anterior spinal cord white matter from WT (littermate) and Cx30 KO EAE mice in the acute (Day 13) and chronic (Day 59) EAE phases. **(D,E)** Confocal images showing immunostaining for Iba-1 and CD3 in spinal cord sections from WT (littermate) and Cx30 KO EAE mice in the acute (Day 13) and chronic (Day 59) EAE phases. Scale bars, 200 μm. Higher magnification images show co-labeling of CD3 and Iba-1 in spinal cords, which are highlighted by a white rectangular frame in the lower magnification images above. **(F)** Quantification of the CD3^+^ cell area fraction in the anterior spinal cords from WT (littermate) and Cx30 KO EAE mice in the acute (Day 13) and chronic (Day 59) EAE phases. **(G,H)** Quantification of the Iba-1^+^ cell area fraction **(G)** and microglial circularity **(H)** in the spinal cord white and gray matter from WT (littermate) and Cx30 KO EAE mice in the acute (Day 13) and chronic (Day 59) EAE phase. Means ± S.E.M. are shown. Statistical differences were determined using the unpaired *t*-test with Welch's correction. N.S. = not significant. *n* indicates the number of mice and each scatter dot represents individual mice in each group.

Cx30 KO mice did not show any significant differences in the incidence, onset day, and clinical severity (peak score and acute phase AUC from Day 9 to 24) of acute EAE compared with WT mice, in agreement with a previous study (29) (Figure 4A). By contrast, clinical severity in the chronic EAE phase (chronic phase AUC from Day 25 to 59) was significantly attenuated in Cx30 KO mice compared with WT mice. HE staining showed that the infiltration of inflammatory cells into the spinal cord was markedly reduced in Cx30 KO mice compared with WT mice in the chronic EAE phase (Figure 4B). Moreover, the extent of demyelination was significantly decreased in the chronic but not acute phase of EAE in Cx30 KO mice compared with WT mice, in both the anterior and posterior parts of the spinal cord (*p* = 0.0031 and *p* = 0.002, respectively, by Fluoromyelin staining; and *p* = 0.0328 and *p* = 0.0167, respectively, by MBP immunostaining; Figures 4C–E, Supplementary Figures 2E,F). CD45^+^ immunocytes and CD3^+^ T cells were not significantly different between Cx30 KO and WT mice in either acute or chronic phases, although CD45^+^ cells tended to be increased in Cx30 KO mice compared with WT mice at acute phase (*p* = 0.0622; Figures 4C,D,F). We performed CD169 immunostaining to discriminate peripheral blood-borne macrophages from microglia and other immune cells, and found that CD169^+^ macrophages were significantly lower in Cx30 KO mice compared with WT mice in the chronic phase (*p* = 0.0336), but not the acute phase (Figures 5A–C).

### Microglia in Cx30 KO mice are widespread and highly activated in the chronic phase of EAE

Intriguingly, Iba-1^+^ microglial cell numbers were consistently greater in both the white and gray matter of the spinal cord in Cx30 KO mice compared with WT mice in the acute and chronic phases (white matter, *p* = 0.0106 on Day 13 and *p* = 0.0274 on Day 59; gray matter, *p* = 0.0483 on Day 13 and *p* = 0.0111 on Day 59; Figures 5D,E,G). The above-mentioned increased tendency of CD45^+^ cells in the acute phase in Cx30 KO mice might be explained by the earlier and stronger increase of Iba-1^+^ cells compared with WT mice. In the spinal cord white matter, microglial circularity was significantly greater in Cx30 KO mice than in WT mice in both the acute (*p* = 0.045) and chronic phases (*p* = 0.0418; Figure 5H). This increase in Iba-1^+^ cells in the chronic phase of EAE was also observed in the optic nerve (*p* = 0.0067) and cerebellum (*p* = 0.0312) of Cx30 KO mice (Supplementary Figures 4A,B,D,E). In the cerebellum, microglial circularity was significantly greater (*p* = 0.0121) in Cx30 KO mice than in WT mice but there was no significant increase of microglial circularity in the optic nerve (Supplementary Figures 4C,F). These findings suggest that a widespread increase and activation of Iba-1^+^ microglia in inflamed CNS tissues, especially the spinal cord white matter, is a characteristic feature of Cx30 KO mice.

### Microglia in Cx30 KO EAE mice have an anti-inflammatory phenotype in the chronic phase of EAE by gene expression microarrays

To further characterize the activated microglial phenotype in the chronic phase of EAE in Cx30 KO mice, we used MOG_35−55_-induced EAE and isolated microglia from the spinal cords and brains of Cx30 KO and WT mice at Day 39 after immunization, when clinical scores and AUC in the chronic phase were significantly lower in Cx30 KO mice than in WT mice (*p* = 0.0023). Microglia isolated from Cx30 KO EAE spinal cords and brains had lower expression levels of pro-inflammatory genes, such as *IL-1b, Nos2, Tnf*, and *Ptgs2*, but no significant changes in anti-inflammatory gene levels, except for an increase in the *Mrc1*gene (Figure 3F). GSEA analysis revealed that microglia from Cx30 KO mouse spinal cord and brain had less pro-inflammatory gene expressions than those from WT mice in the chronic EAE phase (spinal cord, ES = 0.721, normalized *p* = 0.007, FDR = 0.018; brain, pro-inflammatory genes; ES = 0.643, normalized *p* = 0.016, FDR = 0.037; Table 1 and Figures 3G–J). Furthermore, gene expressions of cytokines/chemokines, complements, alarmins, ROS, MHC, and tumor antigens in spinal cord but not brain microglia were significantly less in Cx30 KO than in WT mice in the chronic EAE phase (Table 1 and Supplementary Figures 3B,D). These findings indicate that the increased numbers of activated microglia in Cx30 KO mice have a reduced pro-inflammatory phenotype, especially in the spinal cord, in the chronic EAE phase.

### Cx30 KO mice upregulate arginase-1 and BDNF but downregulate NOS2 in the chronic phase of EAE

**Figure 6:**
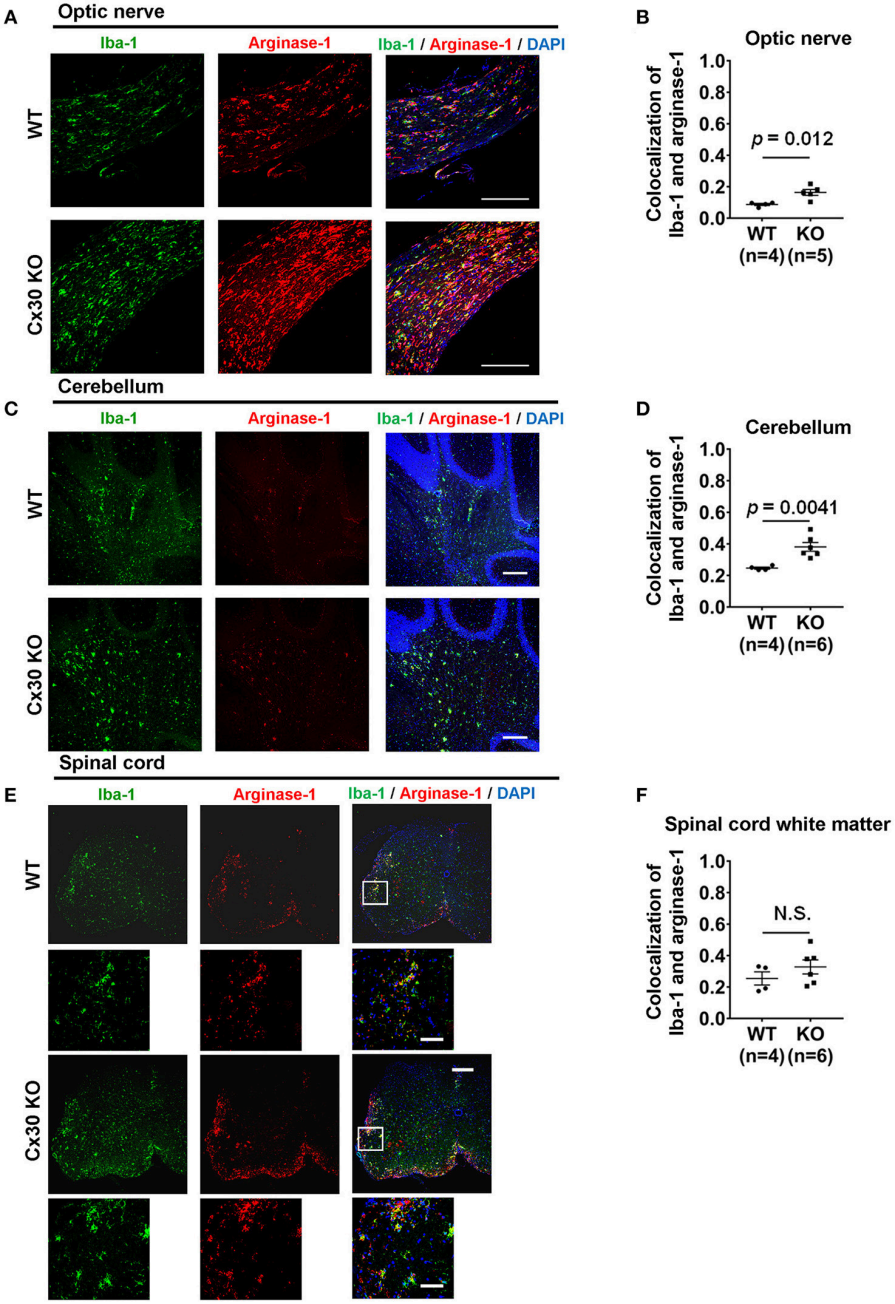
Anti-inflammatory marker expression of microglia in WT and Cx30 KO mice with chronic EAE. **(A,C,E)** Confocal images showing immunostaining for Iba-1 and arginase-1 in optic nerves **(A)**, cerebellum **(C)**, and spinal cords **(E)** from WT (littermate) and Cx30 KO mice in the chronic EAE phase (Day 59). Scale bars, 200 μm. Each higher magnification image is from the white rectangular frame in the lower magnification image above. Scale bars in higher magnification images, 50 μm. **(B,D,F)** Colocalization analysis of arginase-1 and Iba-1 in optic nerves **(B)**, cerebellum **(D)**, and spinal cord white matter **(F)** from WT (littermate) and Cx30 KO mice in the chronic EAE phase, expressed as Pearson's correlation coefficients. Means ± S.E.M. are shown. Statistical differences were determined using the unpaired *t-*test with Welch's correction. N.S. = not significant. *n* indicates number of mice and each scatter dot represents individual mice in each group.

To confirm the anti-inflammatory nature of the activated microglia in the CNS tissues of Cx30 KO mice in the chronic EAE phase, we performed double-staining for Iba-1 and arginase-1, an anti-inflammatory gene. Colocalization of Iba-1 and arginase-1 was more frequently observed in the optic nerve and cerebellum of Cx30 KO mice compared with WT littermate mice (*p* = 0.012, and *p* = 0.0041, respectively; determined by higher mean Pearson's coefficient values; Figures 6A–D), although differences in colocalization levels between WT and Cx30 KO mice did not reach statistical significance in the spinal cord, possibly because of the infiltration of peripheral blood-borne macrophages (Figures 6E,F). Furthermore, in the optic nerve of Cx30 KO mice, BDNF immunoreactivity was significantly greater compared with WT mice (*p* = 0.0019; Supplementary Figures 5A,B). By contrast, NOS2 immunoreactivity in the optic nerve, cerebellum, and spinal cord white matter was significantly lower in Cx30 KO mice than in WT mice (*p* = 0.0003, *p* = 0.0032, and *p* = 0.0047, respectively; Supplementary Figures 5C–H). These findings indicate that microglia in Cx30 KO mice tended to have an anti-inflammatory phenotype, which is more evident in the CNS areas where peripheral blood-borne macrophages are rare during chronic EAE.

### Cx30 deficiency induces the earlier and stronger activation of A2 astrocytes during EAE

**Figure 7:**
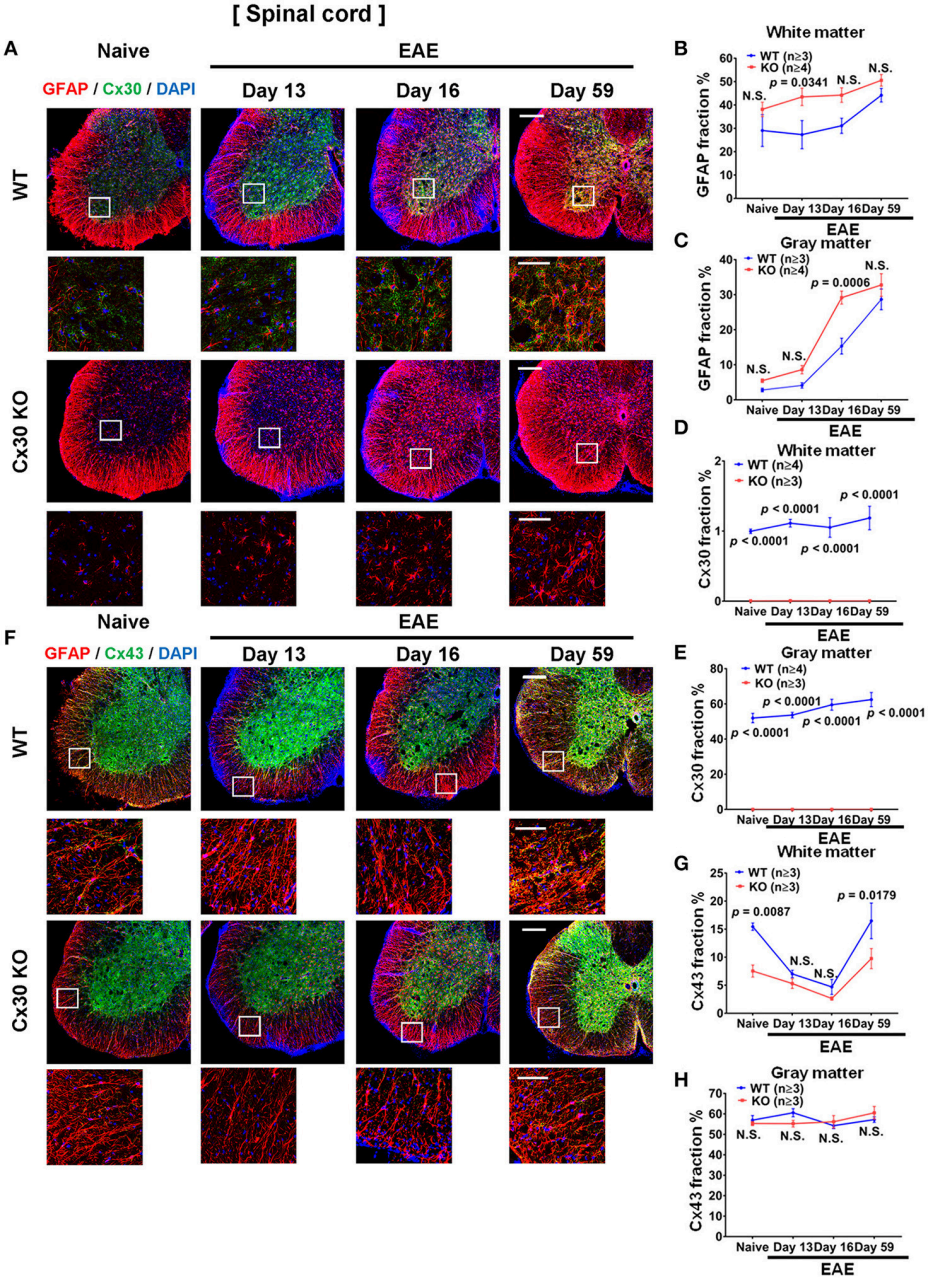
Astrocytic marker expression in spinal cords from WT and Cx30 KO mice during acute and chronic EAE. **(A)** Confocal images showing immunostaining for Cx30 and GFAP in the anterior part of spinal cords from WT (littermate) and Cx30 KO mice in the naïve state and at different stages of EAE. Scale bars, 200 μm. Each higher magnification image is from the white rectangular frame in the lower magnification image above. Scale bars in higher magnification images, 50 μm. **(B,C)** Quantification of the GFAP^+^ cell area fraction in the white **(B)** and gray **(C)** matter. **(D,E)** Quantification of the Cx30^+^ cell area fraction in the white matter **(D)** and gray matter **(E)**. **(F)** Confocal images showing immunostaining for Cx43 and GFAP in the anterior part of spinal cords from WT (littermate) and Cx30 KO mice. Scale bars, 200 μm. Each higher magnification image is from the white rectangular frame in the lower magnification image above. Scale bars in higher magnification images, 50 μm. **(G,H)** Quantification of the Cx43^+^ cell area fraction in the white **(G)** and gray **(H)** matter. Means ± S.E.M. are shown. Statistical differences were determined using two-way ANOVA. N.S. = not significant. *n* indicates number of mice in each group at different stages and each scatter dot represents the mean value for each group.

Compared with naïve spinal cord, numbers of GFAP^+^ astrocytes in the white and gray matter of the spinal cord increased significantly in the chronic EAE phase in both WT and Cx30 KO mice (white matter, *p* = 0.0159 and *p* = 0.0003, respectively; and gray matter, *p* = 0.0008 and *p* = 0.0014, respectively, at Day 59) but not in the acute phase, except for GFAP^+^ astrocytes in the gray matter of Cx30 KO mice at Day 16 (*p* = 0.0154; Figure 7A; Supplementary Figures 6A–D). However, Cx30 KO mice had significantly more GFAP^+^ astrocytes in the spinal cord white and gray matter (white matter; *p* = 0.0341 on Day 13 and gray matter; *p* = 0.0006 on Day 16) during the acute phase compared with WT mice, whereas this difference was not evident in the chronic phase in either the white or gray matter, suggesting the earlier and stronger activation of astrocytes in Cx30 KO mice (Figures 7A–C).

In WT mice, Cx30 immunoreactivity was unchanged throughout the clinical course of EAE in the spinal white matter, but compared with the naïve state it was increased significantly in the spinal cord gray matter in the chronic stage (*p* = 0.0327; Figures 7A,D,E; Supplementary Figures 6E,F). In Cx30 KO mice, Cx30 immunoreactivity was not detected at any stage of EAE (Figures 7A,D,E). By contrast, Cx43 in the spinal cord white matter demonstrated a dynamic change during EAE in WT mice. Cx43 decreased significantly in the acute phase compared with the naïve state (*p* = 0.0149 on Day 13 and *p* = 0.0067 on Day 16) and then recovered to similar levels to the naïve state (significant increase compared with the peak day levels, *p* = 0.0147; Figures 7F,G; Supplementary Figure 6G). A similar decrease of Cx43 in the acute phase and recovery of Cx43 in the chronic phase were also observed in Cx30 KO mice (*p* = 0.0142 at Day 16 and *p* = 0.0026 at Day 59 compared with Day 16; Figures 7F,G; Supplementary Figure 6I); however, Cx43 levels were significantly lower in Cx30 KO mice than in WT mice, in the naïve state and in the chronic EAE phase (*p* = 0.0087 and *p* = 0.0179, respectively) (Figures 7F,G). Cx43 expression in the gray matter did not show obvious changes in either WT or Cx30 KO mice (Figures 7F,H; Supplementary Figures 6H,J).

**Figure 8:**
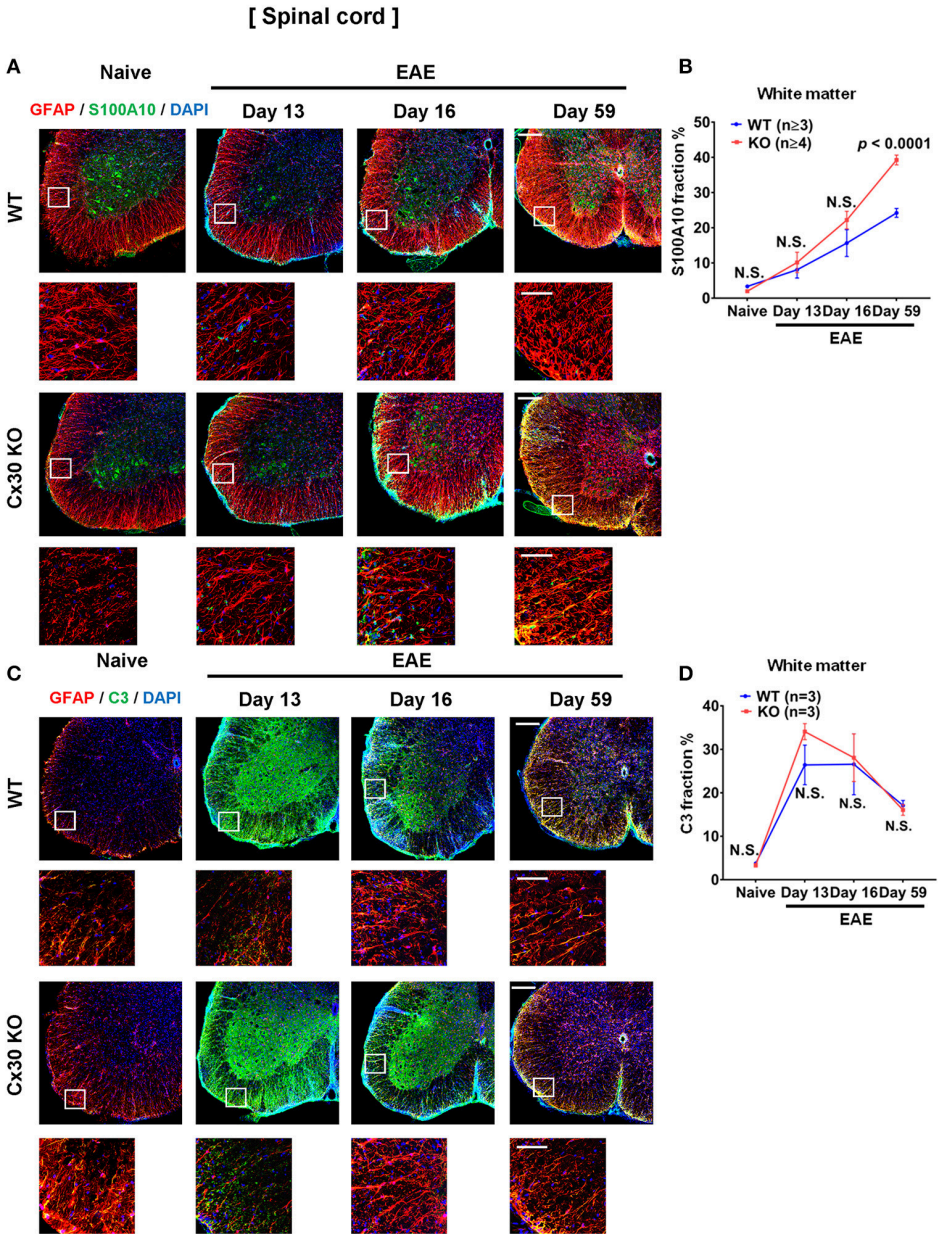
A1 and A2 astrocyte marker expression in spinal cords from WT and Cx30 KO mice during acute and chronic EAE. **(A)** Confocal images showing immunostaining for S100A10 and GFAP in spinal cords from WT (littermate) and Cx30 KO mice. Scale bars, 200 μm. Each higher magnification image is from the white rectangular frame in the lower magnification image above. Scale bars in higher magnification images, 50 μm. **(B)** Quantification of the S100A10^+^ cell fraction in the spinal cord white matter. **(C)** Confocal images showing immunostaining for GFAP and C3 in spinal cords from WT (littermate) and Cx30 KO mice. Scale bars, 200 μm. **(D)** Quantification of the C3^+^ cell fraction in the spinal cord white matter. Each higher magnification image is from the white rectangular frame in the lower magnification image above. Scale bars in higher magnification images, 50 μm. Means ± S.E.M. are shown. Statistical differences were determined using two-way ANOVA. N.S. = not significant. *n* indicates number of mice in each group at different stages and each scatter dot represents the mean value for each group.

We examined A1 and A2 astrocyte markers based on a recent report (53). Immunostaining analyses for S100A10, a representative A2 astrocyte marker, revealed a successive increase in the spinal cord white matter during the course of EAE, which was highest in the chronic EAE stage, in both WT and Cx30 KO mice compared with the naïve state (not significant in either mouse strain on Day 13; not significant and *p* = 0.0251, respectively, on Day 16; *p* = 0.0010 and *p* = 0.0002, respectively, on Day 59; Figures 8A,B; Supplementary Figures 6K,L). Interestingly, this increase in S100A10 immunoreactivity was greater in Cx30 KO mice than in WT mice at all stages of EAE and the difference increased in the later stages of EAE (*p* < 0.0001 at Day 59; Figures 8A,B). By contrast, C3, an A1 astrocyte marker, sharply increased and peaked in the acute phase and then steadily decreased in the chronic phase compared with the naïve state in both WT and Cx30 KO mice (*p* = 0.0174 on Day 13, and *p* = 0.0174 on Day 16, respectively; Figures 8C,D; Supplementary Figures 6M,N). These findings suggest that the stronger activation of A2 astrocytes in Cx30 KO mice plays a role in inducing neuroprotective microglia, which attenuate EAE in the chronic phase.

### Cx30 deficiency causes less neuronal death in the chronic phase of EAE

**Figure 9:**
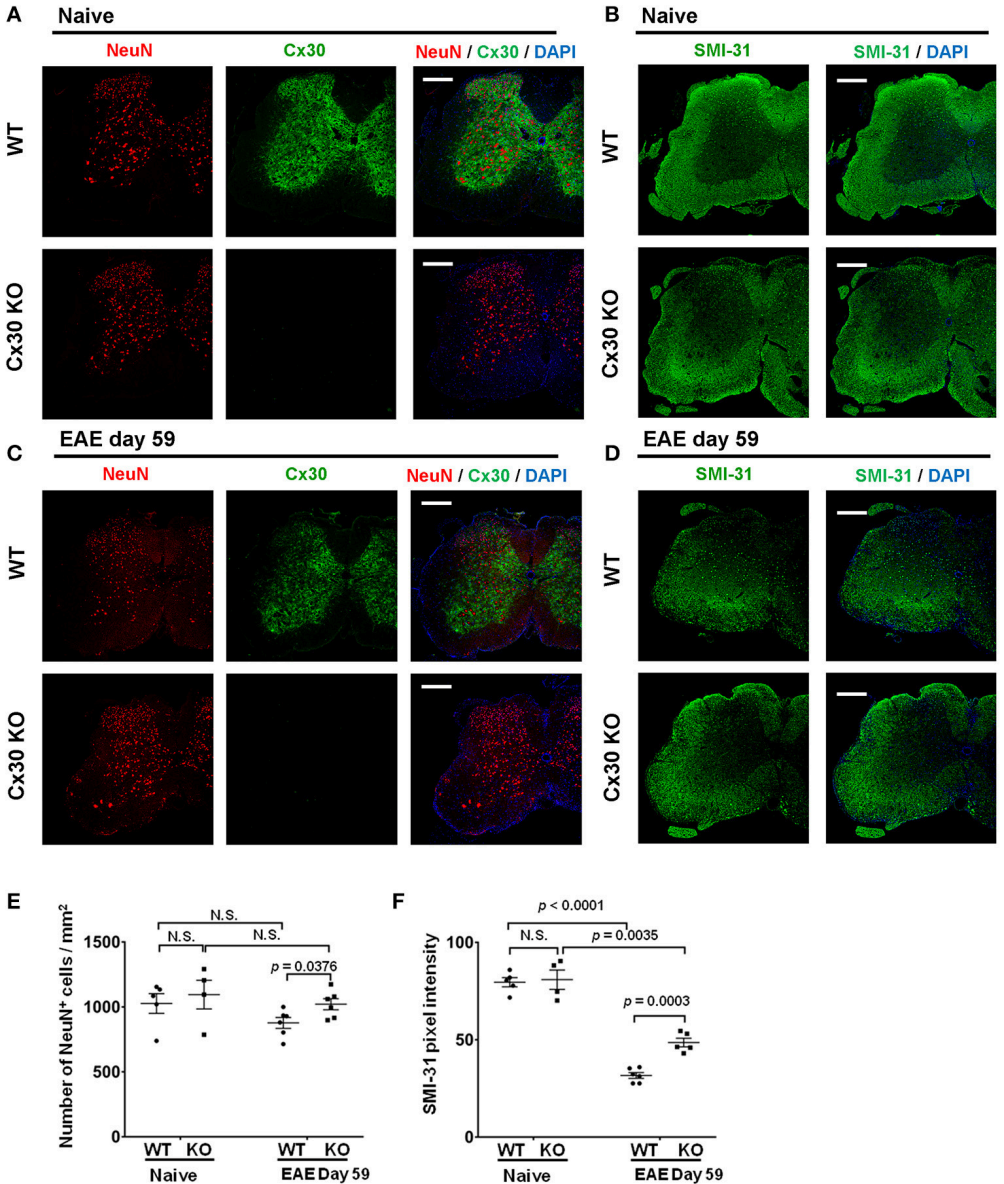
NeuN and SMI-31 immunostaining of spinal cords from WT and Cx30 KO mice with or without EAE. **(A,C)** Confocal images showing immunostaining for Cx30 and NeuN in spinal cords from WT (littermate) and Cx30 KO mice in the naive state **(A)** and chronic EAE phase (Day 59) **(C)**. Scale bars, 200 μm. **(B,D)** Confocal images showing immunostaining for SMI-31 in spinal cord sections from WT (littermate) and Cx30 KO EAE mice in the naive state **(B)** and chronic EAE phase (Day 59) **(D)**. Scale bars, 200 μm. **(E)** Quantification of NeuN^+^ cell numbers/mm^2^ of spinal cord in the naive state or chronic EAE phase. **(F)** Quantification of SMI-31 density in anterior white matter regions of the spinal cord from WT (littermate) and Cx30 KO EAE mice in the naive state and chronic EAE phase (Day 59). Means ± S.E.M. are shown. Statistical differences were determined using the unpaired *t*-test with Welch's correction. N.S. = not significant. *n* indicates the number of mice and each scatter dot represents individual mice in each group.

Finally, because astrocytic Cx30 has close contact with neurons, we examined changes in neurons and axons in Cx30 KO mice during EAE. In the native state, the numbers of NeuN^+^ cells and SMI-31^+^ axonal density were not significantly different between Cx30 and WT littermate mice (Figures 9A–D). Although only SMI-31^+^ axonal density decreased significantly in the chronic EAE phase compared with the naïve state in both WT and Cx30 KO mice (*p* < 0.0001, and *p* = 0.0035, respectively), Cx30 KO mice had significantly more NeuN^+^ cells and SMI-31^+^ remaining axons compared with WT mice in the chronic EAE phase (*p* = 0.0376, and *p* = 0.0003, respectively; Figures 9E,F). Because IL-34, expressed on CNS neurons, induces the differentiation of microglia to a neuroprotective phenotype (54), we examined its expression by immunostaining, and detected significantly more IL-34 in the spinal white matter of Cx30 KO mice than in WT mice in the chronic EAE phase (Supplementary Figure 7). These findings suggest less neuronal death and axonal loss in Cx30 KO mice compared with WT mice in the chronic phase of EAE, leading to more IL-34 production in Cx30 KO mice.

## Discussion

The main new findings in the present study are as follows: (1) Cx30 deficiency attenuated only chronic EAE clinically and pathologically without affecting T cell infiltration. (2) Cx30 deficiency increased the numbers of ramified microglia in the naïve state and induced earlier and more widespread activation of microglia in the acute and chronic phases of EAE. (3) These activated microglia in Cx30 KO mice were prone to differentiate toward an anti-inflammatory phenotype with less pro-inflammatory gene expression. (4) In the naïve state, Cx30 deficiency induced only a modest enlargement of astrocytic processes in the spinal gray matter and a partial reduction of Cx43 expression in the spinal white matter, whereas it caused earlier and stronger activation of astrocytes in the acute EAE phase, upregulated S100A10, a representative A2 astrocyte marker, and attenuated the recovery of Cx43 in the chronic phase. (5) Cx30 deficiency rescued more neurons and axons in the chronic EAE phase without influencing the quantity of neurons or axons in the naïve state.

According to the present study, Cx30 deficiency has no apparent influence on the clinical and histological severity of acute EAE, in accordance with a previous report describing that a single or double KO of Cx30 and/or Cx43 did not affect acute EAE (29). Collectively, this suggests that Cx30 does not modulate the peripheral immune system or alter the clinical course of acute EAE. Surprisingly, chronic EAE and demyelination were significantly attenuated by Cx30 deficiency. These differences in results between a previous study (29) and the current study might be attributable to a difference in the observation period. Thus, acute and chronic EAE are differentially regulated, at least in part, and Cx30 is mainly involved in the chronic phase, when microglia and astrocytes are postulated to be key players (55, 56).

We demonstrated the attenuation of chronic EAE by Cx30 deficiency without influencing T cell infiltration, which further underlines the importance of microglia and astrocytes in the chronic phase of EAE. In naïve Cx30 KO mice, increased microglia were observed throughout the CNS; however, these microglia retained a ramified morphology without a significant increase in circularity. Consistent with this morphology, they had similar expression levels of pro-inflammatory genes to WT microglia by RNA microarray. During the chronic EAE phase in Cx30 KO mice, there were increased numbers and more activated microglia in the spinal cord and the brain, which had reduced pro-inflammatory gene expression compared with WT mice. Indeed, microglia in Cx30 KO mice had lower expressions of *IL-1b, Nos2, Tnf*, and *Ptgs2* and a higher expression of *Mrc1*. IL-1β, tumor necrosis factor-α, NOS2, and prostaglandin-endoperoxide synthase-2, also known as cyclooxygenase-2, are well-known pro-inflammatory molecules involved in neuroinflammation (57–61). Mannose receptor C-type 1 (Mrc1), downregulated by IFN-γ (62), and upregulated by IL-4 (63), is expressed at high levels during the resolution of inflammation where it has a critical role in the removal of inflammatory glycoproteins (64). We also immunohistochemically observed the increased expression of arginase-1 and BDNF and the decreased expression of NOS2 in the optic nerve, cerebrum, and cerebellum. Although such changes were not clear in inflamed spinal cord lesions, the increased infiltration of peripheral blood-borne pro-inflammatory macrophages, reported to be abundant in chronic EAE (65) and shown as a significant increase of CD169^+^ cells in the chronic EAE phase in the present study, might have obscured the reduced pro-inflammatory nature of Cx30 KO mouse spinal cord microglia.

Notably, spinal cord but not brain microglia had significantly lower expressions of cytokines/chemokines, alarmins, and MHC genes in Cx30 KO than in WT mice, suggesting spinal cord microglia are less reactive to inflammatory insults than brain microglia in the naïve state in Cx30 KO mice. In the chronic EAE phase, cytokines/chemokines, complements, alarmins, ROS, and MHC gene expressions in microglia were significantly lower in Cx30 KO than in WT mice in the spinal cord but not in the brain, indicating that spinal cord microglia have a tendency to have a reduced pro-inflammatory and increased anti-inflammatory phenotype compared with brain microglia in Cx30 KO mice.

Earlier and stronger activation of astrocytes in EAE was another characteristic feature of Cx30 KO mice. S100A10, a neuroprotective A2 astrocyte-related gene product (53), was increasingly expressed in the spinal cord white matter as EAE proceeded from the acute to chronic phase. Although the precise functions of astrocytic S100A10 remain to be elucidated, it was reported to be required for membrane repair (66), cell proliferation (67), and inhibition of cell apoptosis by interaction with Bcl-xL/Bcl-2-associated death promoter (68). Thus, S100A10 expressed in astrocytes might be beneficial for tissue repair. These findings collectively suggest that Cx30-deficient astrocytes may be prone to differentiate toward an A2 astrocyte phenotype upon activation. In addition, these Cx30-deficient astrocytes showed less Cx43 immunoreactivity in the astrogliotic scar in the chronic EAE phase as well as in the naïve state. The overexpression of Cx43 might propagate inflammatory mediators through homotypic Cx43 GJ channels and Cx43 hemichannels (69–72). Thus, the downregulation of Cx43 on Cx30-deficient astrocytes may also function in suppressing chronic neuroinflammation.

Increased numbers of GFAP^+^ astrocytes were present in the white matter compared with the gray matter of Cx30 KO mice spinal cords. Furthermore, increased S100A10 and decreased Cx43 levels were observed in the spinal white matter, suggesting white matter astrocytes may exhibit earlier and stronger activation toward an A2 phenotype than gray matter astrocytes in the Cx30 KO spinal cord. Because increased microglia numbers were observed in spinal white and gray matter in Cx30 KO mice while a circularity increase was detected only in white matter microglia, the activation of microglia toward an anti-inflammatory phenotype may occur more strongly in the spinal white matter than in the gray matter. Collectively, earlier and stronger astroglial activation toward an A2 phenotype occurred in the spinal white matter, which might partly explain the induction of anti-inflammatory microglia in Cx30 KO mice spinal white matter. Such microglial activation occurred even in the acute EAE phase in Cx30 KO mice; however, this effect might not be sufficient in the acute phase when CD3^+^ T cells mainly induce acute inflammation, while becoming evident in the chronic phase when glial inflammation predominates.

Interestingly, Cx30 KO mice had more remaining neurons and axons than WT mice in the chronic phase of EAE. Cx30 on astrocytes exists in close proximity to neurons (32) and Cx30 hemichannels release glutamate that exerts excitotoxicity on neurons (54, 73). Thus, Cx30 deficiency may confer survival of neurons and axons. Neuronal IL-34 is a potent activator for microglia toward a neuroprotective phenotype (74). Accordingly, we hypothesized that the earlier and stronger activation of astroglia toward an A2 phenotype in Cx30 KO mice in the spinal white matter induced anti-inflammatory microglia (75, 76). Increased axonal IL-34 in Cx30 KO mice may also contribute to the induction of anti-inflammatory microglia in the chronic EAE phase (74), which might dampen chronic neuroinflammation.

In conclusion, Cx30 on astrocytes plays a significant role in perpetuating neuroinflammation in chronic EAE by augmenting pro-inflammatory glial responses. How Cx30 deficiency attenuates inflammatory glial responses should be investigated in future studies, and might provide valuable information for new therapeutic strategies for chronic glial inflammation in MS.

## Author contributions

MF, RY, GL, KM, HY, and AF performed experiments. MF, RY, HY, and JK designed the research. MF, RY, KM, NI, and JK analyzed the data and provided scientific suggestions. MF, RY, and JK drafted the manuscript. All authors reviewed the manuscript.

### Conflict of interest statement

RY has received honoraria from Biogen Japan and Japan Blood Products Organization; Takeda Pharmaceutical Co. Ltd., and Mitsubishi Tanabe Pharma; JK is a consultant for Biogen Japan and Medical Review and has received honoraria from Bayer Healthcare, Mitsubishi Tanabe Pharma, Nobelpharma, Otsuka Pharmaceutical, Sanofi K.K., Chugai Pharmaceutical Co. Ltd., Teijin Pharma, Novartis Pharma, and Medical Review. The remaining authors declare that the research was conducted in the absence of any commercial or financial relationships that could be construed as a potential conflict of interest.
