# Supplementary material for: Connexin 30 Deficiency Attenuates Chronic but Not Acute Phases of Experimental Autoimmune Encephalomyelitis Through Induction of Neuroprotective Microglia
Source: Front Immunol. 2018 Nov 7;9:2588. doi: 10.3389/fimmu.2018.02588 (PMC6234958; doi:10.3389/fimmu.2018.02588)
Supplement: Supplementary file 1 [file Data_Sheet_1.PDF]

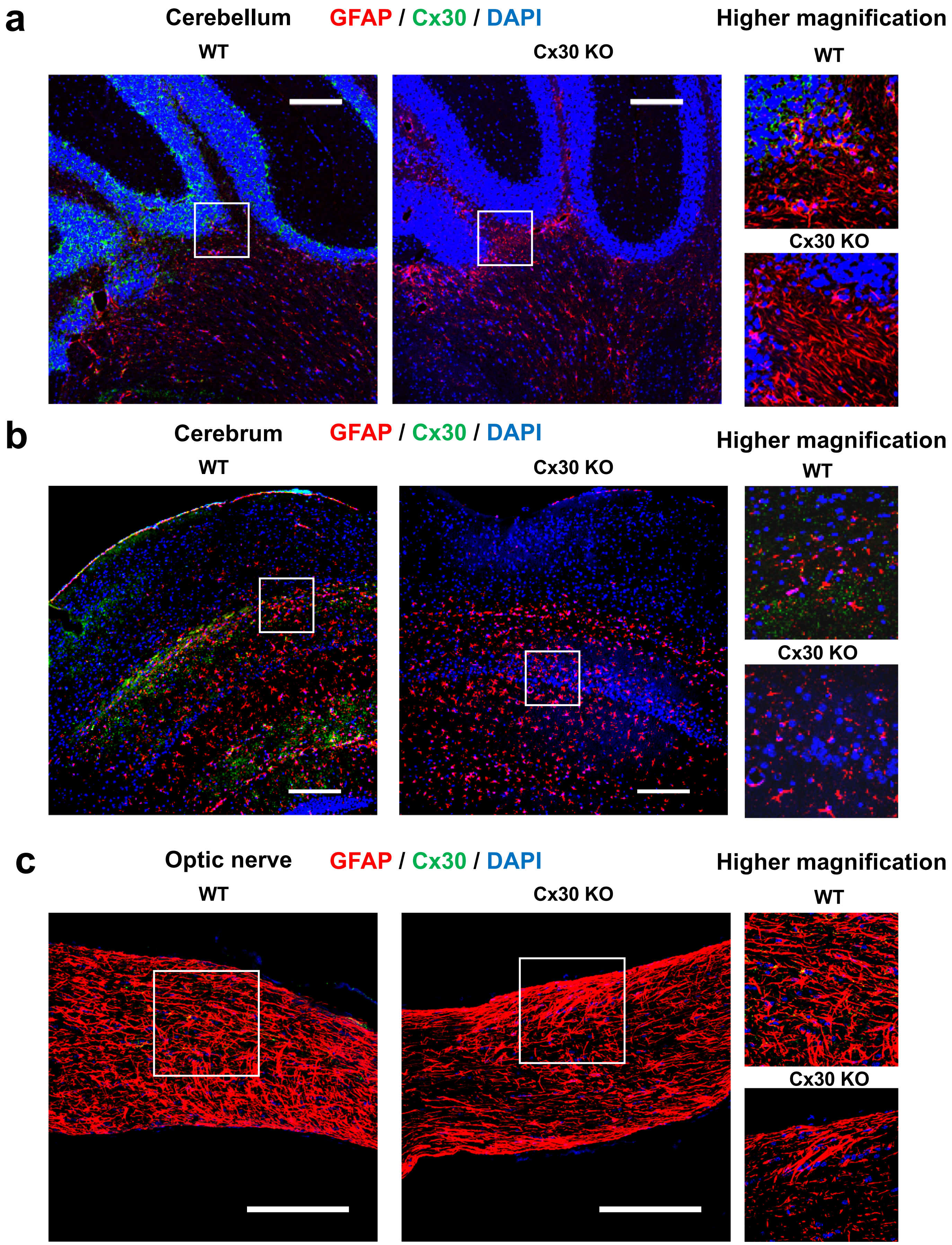

Supplementary Figure 1. Cx30 immunostaining of the cerebellum, cerebrum, and optic nerve. Confocal images showing immunostaining for Cx30 and GFAP in the cerebellum (**a**), cerebrum (**b**), and optic nerve (**c**) of naïve WT (littermate) and Cx30 KO mice. Higher magnification images show co-labeling of GFAP and Cx30 highlighted by a white square in each figure. Scale bars, 200 μm.

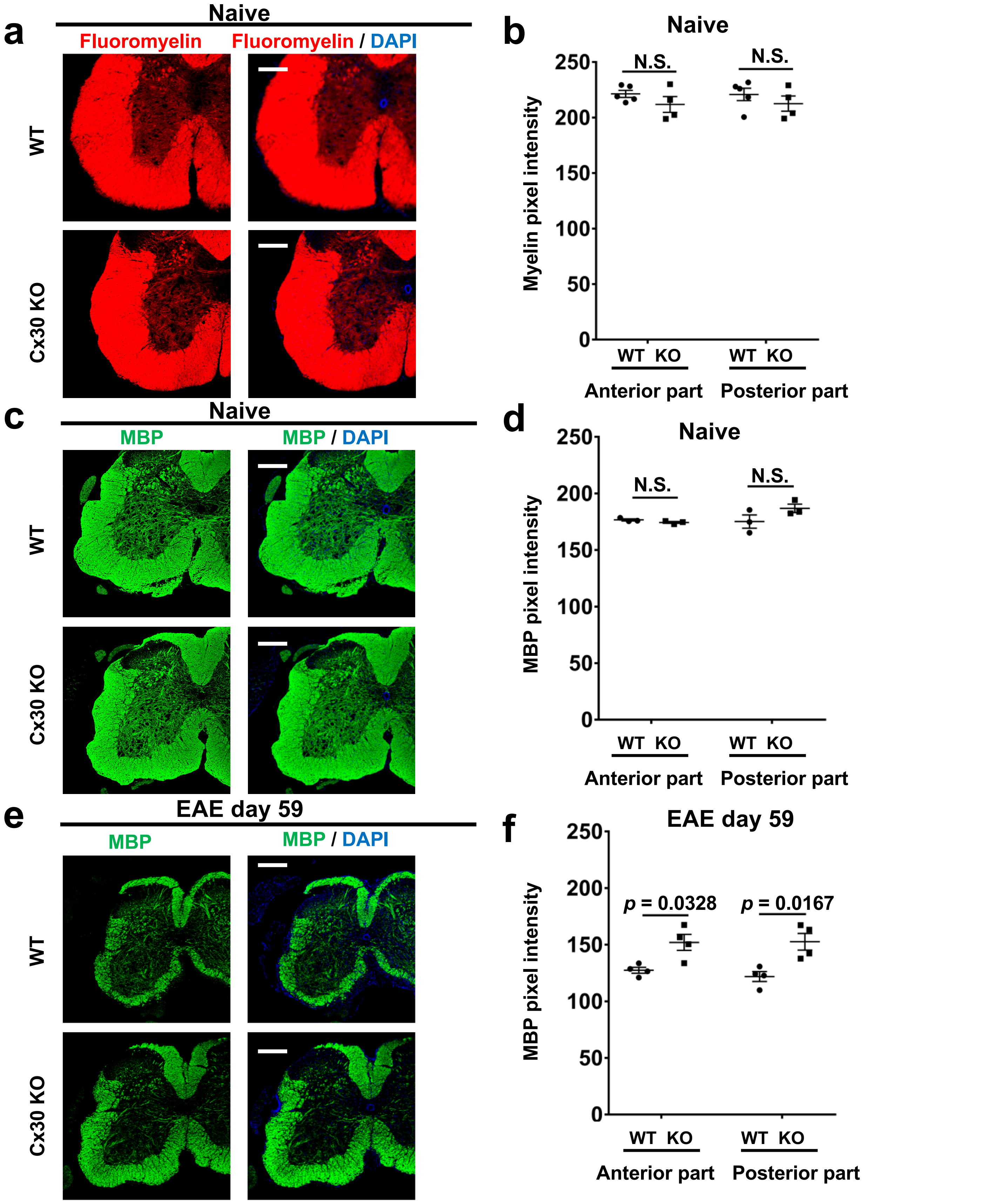

Supplementary Figure 2. Fluoromyelin and MBP immunostaining of spinal cords.
(**a**, **c**) Confocal images showing immunostaining for Fluoromyelin (**a**) and MBP (**c**) in spinal cords from naive WT (littermate) and Cx30 KO mice. Scale bars, 200 μm. (**b**, **d**) Quantification of myelin (**b**) and MBP (**d**) density in the anterior and posterior parts of spinal cords from naive WT (littermate) and Cx30 KO mice. (**e**) Confocal images showing immunostaining for MBP in spinal cords from WT (littermate) and Cx30 KO mice in the chronic EAE phase (Day 59). Scale bars, 200 μm. (**f**) Quantification of MBP density in the anterior and posterior parts of spinal cords from WT (littermate) and Cx30 KO mice in the chronic EAE phase (Day 59). Means ± S.E.M. are shown. Statistical differences were determined using the unpaired *t*-test with Welch's correction. N.S. = not significant. *n* indicates the number of mice and each scatter dot represents individual mice in each group.

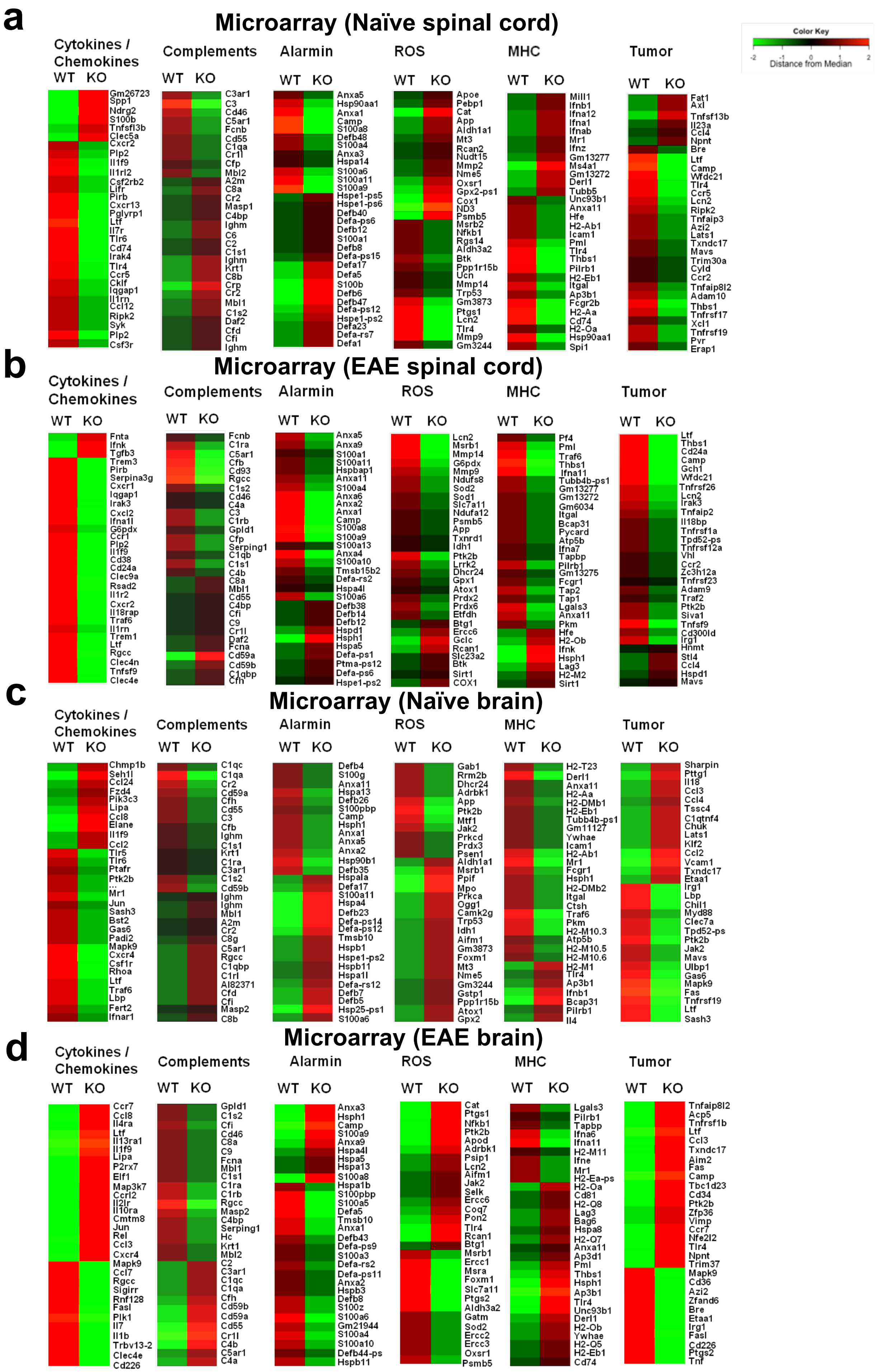

Supplementary Figure 3. Microarray analysis of microglia from spinal cords and brains of WT and Cx30 KO mice. Cluster analysis of gene expression arrays classified into six common pathways: cytokines/chemokines, complements, alarmins, reactive oxygen species, major histocompatibility complex antigens, and tumor-related genes from naive spinal cords (**a**) and brains (**c**), and chronic EAE spinal cords (**b**) and brains (**d**) of WT (C57BL/6) and Cx30 KO mice.

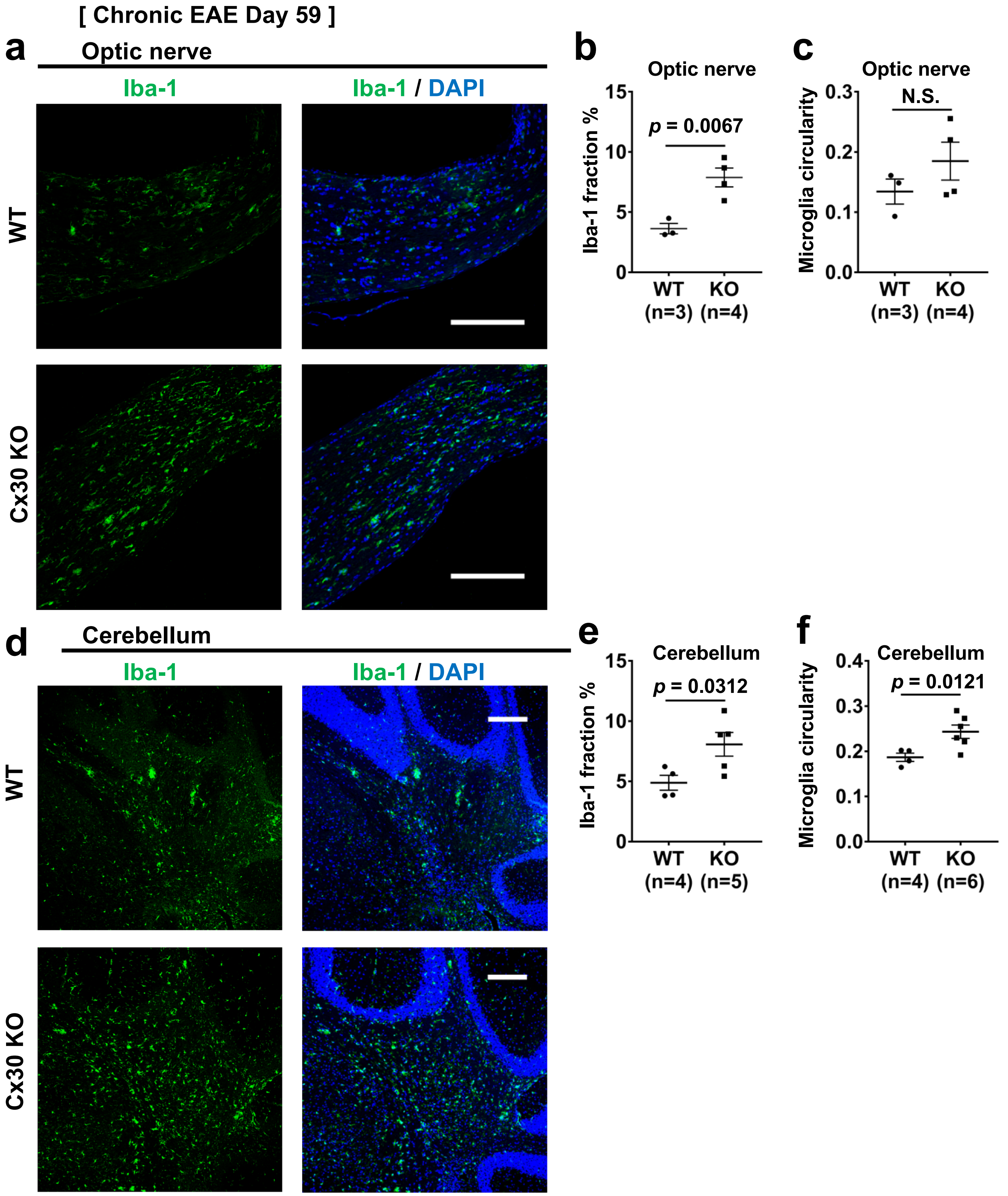

Supplementary Figure 4. Morphology and number of microglia in the optic nerve and cerebellum of WT and Cx30 KO mice with chronic EAE. (**a**, **d**) Confocal images demonstrating immunostaining for Iba-1 in the optic nerve (**a**) and cerebellum (**d**) of WT (littermate) and Cx30 KO EAE mice in the chronic EAE phase (Day 59). Scale bars, 200 μm. (**b**, **e**) Quantification of the Iba-1^{+} cell fraction in the optic nerve (**b**) and cerebellum (**e**) of WT (littermate) and Cx30 KO EAE mice in the chronic EAE phase (Day 59). (**c**, **f**) Quantification of microglial circularity in the optic nerve (**c**) and cerebellum (**f**) of WT (littermate) and Cx30 KO EAE mice in the chronic EAE phase (Day 59). Means ± S.E.M. are shown. Statistical differences were determined using the unpaired *t*-test with Welch's correction. N.S. = not significant, *n* indicates the number of mice and each scatter dot represents individual mice in each group.

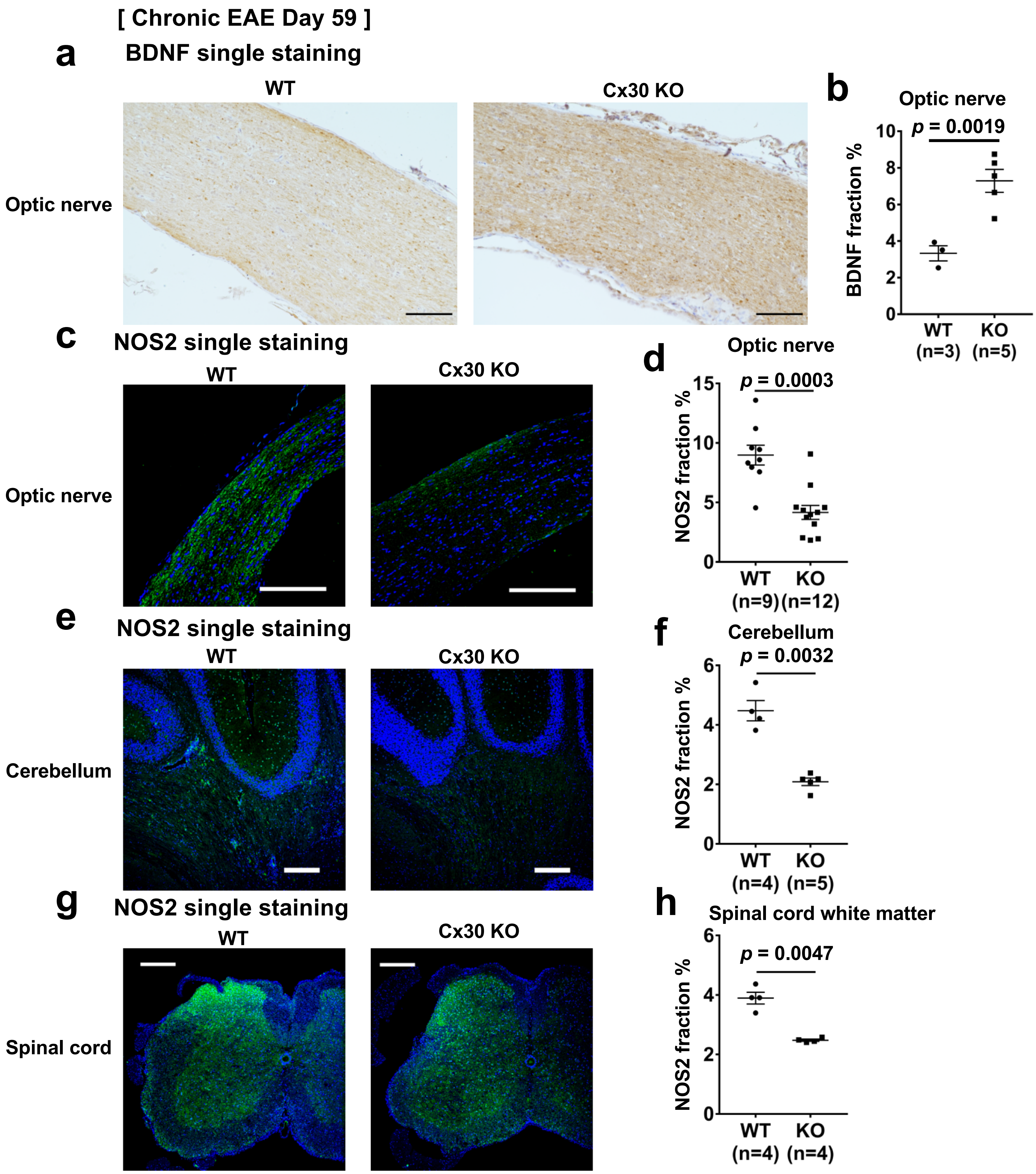

Supplementary Figure 5. BDNF and NOS2 immunostaining in CNS tissues from WT (littermate) and Cx30 KO mice with chronic EAE. (**a**) DAB staining of BDNF in optic nerves from WT and Cx30 KO EAE mice in the chronic EAE phase (Day 59). Scale bars, 100 μm. (**b**) Quantification of the BDNF^{+} cell fraction in spinal cords from WT and Cx30 KO EAE mice in the chronic EAE phase (Day 59). (**c**, **e**, **g**) Representative images of NOS2 and DAPI immunofluorescent staining of the optic nerve (**c**), cerebellum (**e**), and spinal cord (**g**) of WT and Cx30 KO EAE mice in the chronic EAE phase (Day 59). Scale bars, 200 μm. (**d**, **f**, **h**) Quantification of the NOS2^{+} cell fraction in the optic nerve (**d**), cerebellum (**f**), and spinal cord white matter (**g**) of WT and Cx30 KO EAE mice in the chronic EAE phase (Day 59). Means ± S.E.M. are shown. Statistical differences were determined using the unpaired *t*-test with Welch's correction. N.S. = not significant. *n* indicates the number of mice and each scatter dot represents individual mice in each group.

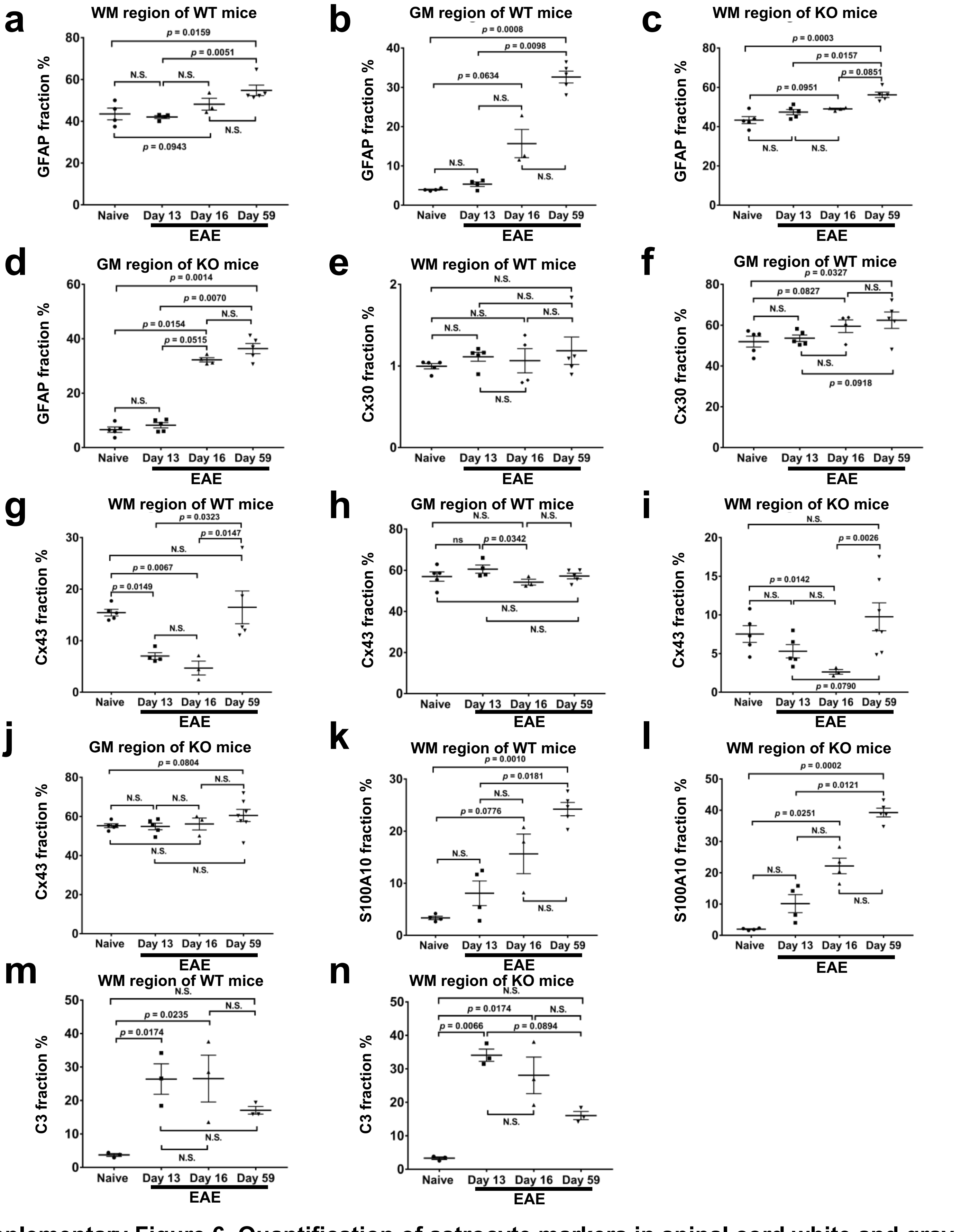

Supplementary Figure 6. Quantification of astrocyte markers in spinal cord white and gray matter from naïve WT and Cx30 KO mice and at different EAE stages (Days 13, 16 and 59). (**a**–**d**) GFAP⁺ cell fractions in the white (**a**) and gray (**b**) matter of WT mice and white (**c**) and gray (**d**) matter of Cx30 KO mice. (**e**, **f**) Cx30⁺ cell fractions in the white (**e**) and gray (**f**) matter of WT mice. (**g**–**j**) Cx43⁺ cell fractions in the white (**g**) and gray (**h**) matter of WT mice and white (**i**) and gray (**j**) matter of Cx30 KO mice. (**k**, **l**) S100A10⁺ cell fractions in the white matter of WT (**k**) and Cx30 KO (**l**) mice. (**m**, **n**) C3⁺ cell fractions in the white matter of WT (**m**) and Cx30 KO (**n**) mice. Means ± S.E.M. are shown. Statistical differences were determined using one-way ANOVA. N.S. = not significant. Each scatter dot represents individual mice at different stages.

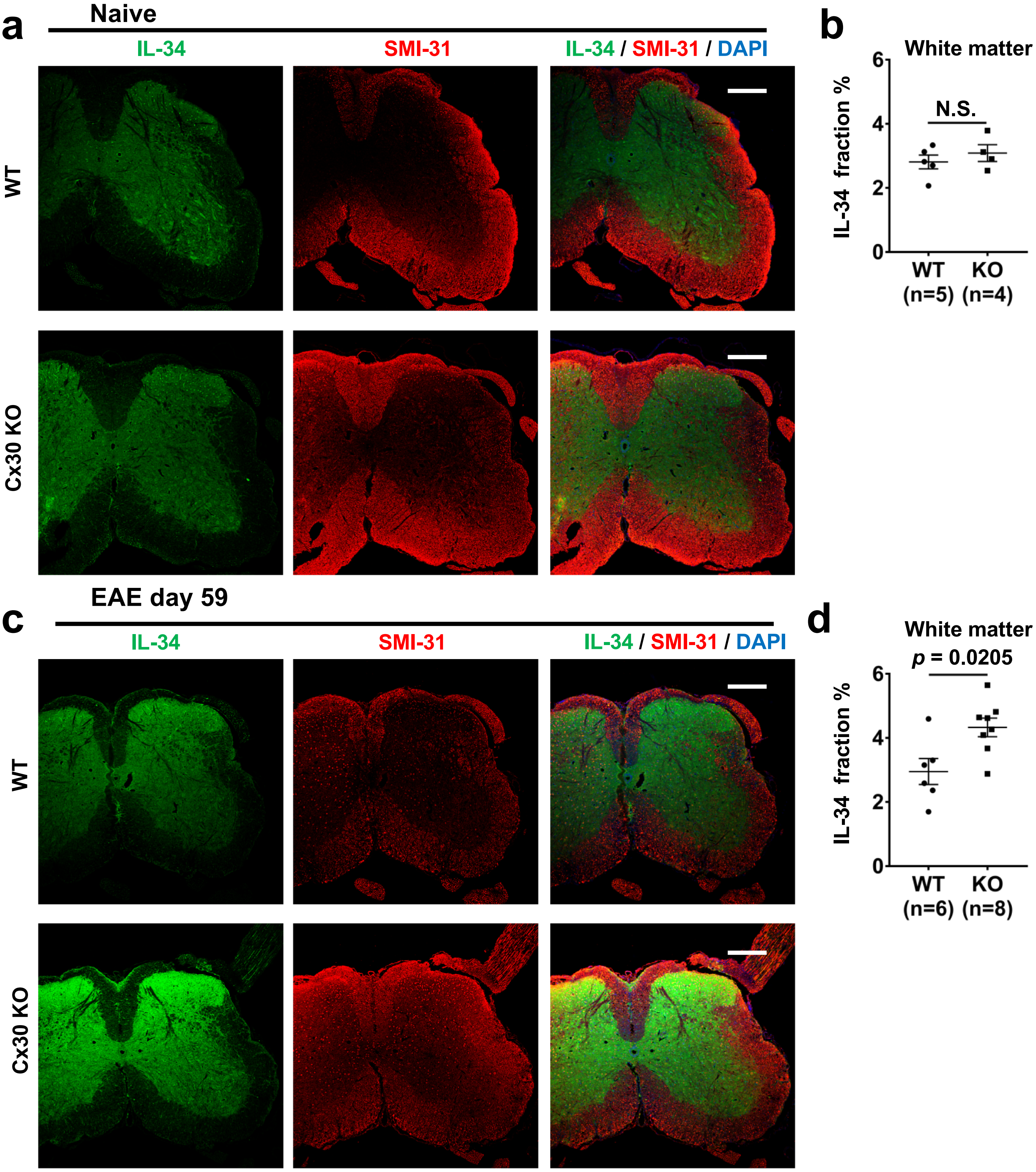

Supplementary Figure 7. IL-34 and SMI-31 immunostaining of spinal cords in WT and Cx30 KO mice with or without EAE. (**a**, **c**) Confocal images showing immunostaining for IL-34 and SMI-31 in spinal cords from WT (littermate) and Cx30 KO mice in the naive state (**a**) and chronic EAE phase (Day 59) (**c**). Scale bars, 200 μm. (**b**, **d**) Quantification of IL-34 positive cell fractions in the white matter of anterior spinal cords in the naive state (**b**) and chronic EAE phase (**d**). Means ± S.E.M. are shown. Statistical differences were determined using the unpaired *t*-test with Welch's correction. N.S. = not significant. *n* indicates the number of mice and each scatter dot represents individual mice in each group.

Supplementary Table 1. Description of antibodies used.

Antibody	Clone	Type	Dilution	Buffer	Antigen retrieval	Incubation condition	Source
Anti-liver-type arginase (arginase 1)	43-81 Monoclonal	Mouse IgG1, κ	1:100	5% NGS, 1% BSA in 50 mM Tris-HCl	Not done	4°C overnight	Santa Cruz Biotechnology
Anti-brain-derived neurotrophic factor (BDNF)	Polyclonal	Rabbit	1:50	5% NGS, 1% BSA in 50 mM Tris-HCl	Autoclave, 10 mM citrate buffer	4°C overnight	Santa Cruz Biotechnology
Anti-complement component C3	11H9 Monoclonal	Rat IgG2a	1:50	10% BSA in PBS-T (0.1% Triton)	Not done	4°C overnight	Hycult Biotech
Anti-connexin 30	16H9L8 Monoclonal	Rabbit IgG	1:500	IHC-P: 5% NGS, 1% BSA in 50 mM Tris-HCl<br>IHC-F: 10% BSA in PBS-T (0.1% Triton)	Not done	4°C overnight	Thermo Fisher Scientific
Anti-connexin 43/GJA1	Polyclonal	Rabbit	1:1,000	10% BSA in PBS-T (0.1% Triton)	Not done	4°C overnight	Abcam
Anti-glial fibrillary acidic protein (GFAP)-Cy3	Monoclonal	Mouse IgG1	1:400	IHC-P: 5% NGS, 1% BSA in 50 mM Tris-HCl<br>IHC-F: 10% BSA in PBS-T (0.1% Triton)	Not done	4°C overnight	Sigma-Aldrich
Anti-glial fibrillary acidic protein (GFAP)	Polyclonal	Rabbit IgG	1:1,000	IHC-P: 5% NGS, 1% BSA in 50 mM Tris-HCl<br>IHC-F: 10% BSA in PBS-T (0.1% Triton)	Not done	4°C overnight	DAKO
Anti-Iba1	Polyclonal	Rabbit IgG	1:1,000	IHC-P: 5% NGS, 1% BSA in 50 mM Tris-HCl<br>IHC-F: 10% BSA in PBS-T (0.1% Triton)	Not done	4°C overnight	Wako
Anti-IL-34	Polyclonal	Rabbit IgG	1:300	IHC-P: 5% NGS, 1% BSA in 50 mM Tris-HCl<br>IHC-F: 10% BSA in PBS-T (0.1% Triton)	Not done	4°C overnight	ProSci Inc
Anti-mouse CD169	Monoclonal	Rat IgG2a, κ	1:100	10% BSA in PBS-T (0.1% Triton)	Not done	4°C overnight	BD Pharmingen
Anti-mouse CD3 molecular complex	17A2 Monoclonal	Rat IgG2b, κ	1:100	10% BSA in PBS-T (0.1% Triton)	Not done	4°C overnight	BD Pharmingen

Anti-myelin basic protein	Polyclonal	Rabbit	1:500	5% NGS, 1% BSA in 50 mM Tris-HCl	Not done	4°C overnight	Abcam
Anti-CD45	IBL-3/16 Monoclonal	Rat IgG1	1:50	10% BSA in PBS-T (0.1% Triton)	Not done	4°C overnight	Bio-Rad AbD Serotec
Anti-nos2/inos	Polyclonal	Rabbit IgG	1:50	5% NGS, 1% BSA in 50 mM Tris-HCl	Autoclave, 10 mM citrate buffer	4°C overnight	Santa Cruz Biotechnology
Anti-S100a10	Polyclonal	Goat IgG	1:100	10% BSA in PBS-T (0.1% Triton)	Not done	4°C overnight	R&D Systems, Inc.
Purified anti-neurofilament H (NF-H), phosphorylated antibody (SMI 31)	SMI 31	Mouse IgG1, κ	1:1,000	5% NGS, 1% BSA in 50 mM Tris-HCl	Not done	4°C overnight	BioLegend
PE/Cy7-conjugated anti-mouse CD11b	M1/70	Rat IgG2b, κ	1:100	1% BSA and 0.1% sodium azide in PBS	Not done	4°C 30 min	BioLegend
PerCP-conjugated anti-mouse CD45	30-F11	Rat IgG2b, κ	1:100	1% BSA and 0.1% sodium azide in PBS	Not done	4°C 30 min	BioLegend
